# Multiple sequences orchestrate subcellular trafficking of neuronal PAS domain–containing protein 4 (NPAS4)

**DOI:** 10.1074/jbc.RA118.001812

**Published:** 2018-06-13

**Authors:** Beata Greb-Markiewicz, Mirosław Zarębski, Andrzej Ożyhar

**Affiliations:** From the ‡ Department of Biochemistry, Faculty of Chemistry, Wrocław University of Science and Technology, Wybrzeże Wyspiańskiego 27, 50–370 Wrocław, Poland and; the § Department of Cell Biophysics, Faculty of Biochemistry, Biophysics, and Biotechnology, Jagiellonian University, Gronostajowa 7, 30-387 Kraków, Poland

**Keywords:** basic helix–loop–helix transcription factor (bHLH), cell culture, intracellular trafficking, microscopic imaging, protein expression, bHLH-PAS, glucose concentration effect, NES, NLS, NPAS4

## Abstract

Neuronal Per-Arnt-Sim (PAS) domain–containing protein 4 (NPAS4) is a basic helix–loop–helix (bHLH)-PAS transcription factor first discovered in neurons in the neuronal layer of the mammalian hippocampus and later discovered in pancreatic β-cells. NPAS4 has been proposed as a therapeutic target not only for depression and neurodegenerative diseases associated with synaptic dysfunction but also for type 2 diabetes and pancreas transplantation. The ability of bHLH-PAS proteins to fulfil their function depends on their intracellular trafficking, which is regulated by specific sequences, *i.e.* the nuclear localization signal (NLS) and the nuclear export signal (NES). However, until now, no study examining the subcellular localization signals of NPAS4 has been published. We show here that *Rattus norvegicus* NPAS4 was not uniformly localized in the nuclei of COS-7 and N2a cells 24 h after transfection. Additionally, cytoplasmic localization of NPAS4 was leptomycin B-sensitive. We demonstrate that NPAS4 possesses a unique arrangement of localization signals. Its bHLH domain contains an overlapping NLS and NES. We observed that its PAS-2 domain contains an NLS, an NES, and a second, proximally located, putative NLS. Moreover, the C terminus of NPAS4 contains two active NESs that overlap with a putative NLS. Our data indicate that glucose concentration could be one of the factors influencing NPAS4 localization. The presence of multiple localization signals and the differentiated localization of NPAS4 suggest a precise, multifactor-dependent regulation of NPAS4 trafficking, potentially crucial for its ability to act as a cellular stress sensor and transcription factor.

## Introduction

Neuronal Per-Arnt-Sim domain–containing protein 4 (NPAS4)^2^ (also known as neuronal transcription factor (NXF), limbic-enriched PAS domain protein (LE-PAS), and PAS domain–containing protein 10 (PASD10)) belongs to the basic helix–loop–helix (bHLH)-Per-Arnt-Sim (PAS) family of transcription factors (1, 2). This family of proteins is well-conserved from metazoans to humans and plays important roles in maintaining cellular health by acting as environmental sensors (3). The bHLH-PAS family is divided into two classes according to the mechanism of dimerization. Class I proteins define the specificity of target gene activation and form functional heterodimers with class II proteins. Aryl hydrocarbon receptors and hypoxia-inducible factors (HIF, HIF-1α, HIF-2α, and HIF-3α) are examples of class I proteins. Class II proteins form homodimers and are general heterodimeric partners for class I bHLH-PAS proteins. Aryl hydrocarbon nuclear translocator (ARNT, also known as HIF-1β), ARNT2, brain and muscle ARNT-like 1 (BMAL1, also known as ARNTL), and BMAL2 (ARNTL2) are examples of class II bHLH-PAS proteins (4, 5). Via a two-hybrid assay, NPAS4 was found to be a class I bHLH-PAS transcription factor forming functional heterodimers with ARNT1, ARNT2, and BMAL1 (1). ARNT1 and ARNT2 were subsequently confirmed as heterodimeric partners of NPAS4 in the brain (6).

NPAS4 was first discovered in mammalian neurons in the neuronal layer of the hippocampus (6) and later detected in nonneuronal tissues. NPAS4 was shown to be highly induced by activity and stress in pancreatic β-cells, to reduce insulin content, to impair responsiveness to glucagon-like peptide 1 (GLP-1), and to protect pancreatic β-cells from ER stress (7). Interestingly, expression of NPAS4 was also documented in human endothelial cells. Esser *et al.* (8) present NPAS4 as a regulator of blood vessel formation and a factor necessary for endothelial cell function such as migration, sprouting, and branch formation in constructing new vasculature.

NPAS4 has been proposed as a novel therapeutic target for depression and neurodegenerative diseases associated with synaptic dysfunction (9), as a component of new stroke therapies (10), as a therapeutic target for diabetes (11), and as a treatment during pancreas transplantation (12). NPAS4 has also been shown to enhance autophagic clearance of the total and phosphorylated pools of endogenous Tau protein in primary cortical neurons, implicating NPAS4 as a potential therapeutic target for neurodegenerative tauopathies such as Alzheimer's disease (13).

Eukaryotic cells possess distinct nuclear and cytoplasmic compartments separated by a double-membrane nuclear envelope with nuclear pore complexes, which allow the exchange of macromolecules between these two compartments (14). To enable transcriptional responses, intra- or extracellular signals need to be transmitted to the nucleus; nucleocytoplasmic shuttling of transcription factors is a mechanism involved in this process (15). Nuclear pore complexes allow passive diffusion of ions and small proteins (<40 kDa) through the nuclear envelope, but passage of larger molecules is restricted to those containing appropriate targeting signals recognized by specific carrier proteins known as karyopherins. Karyopherins engaged in nuclear import are known as importins, and those engaged in export from the nucleus are known as exportins (16). Subcellular localization and shuttling of proteins between nuclear and cytoplasmic compartments are consequences of a dynamic balance between operational strengths of nuclear localization signals (NLS) and nuclear export signals (NES) (14). It has been shown that leptomycin B (LMB) inhibits protein transport from the nucleus to the cytoplasm by interacting with exportin 1, which depends on a leucine-enriched sequence known as the classical NES (17–19).

The subcellular localization of NPAS4 was first reported in a study examining full-length NPAS4 48 h after transfecting COS-7 cells, revealing a strict nuclear localization of this protein (2). Analyses performed in HEK293 cells 48 h after transfection yielded a similar result (20). However, Sullivan *et al.* (21) show by immunofluorescent staining of fixed HEK293T cells 24 h after transfection that NPAS4 even though most staining was nuclear, some staining was also presented in the cytoplasm. Additionally, Western blot analysis of samples obtained by subcellular fractionation of rat coronal brain tissue reveal NPAS4 expression not only in the nucleus but also in the cytoplasm of cells. Ischemic insult resulted in partial translocation of NPAS4 from the cytoplasm to intracellular membranes. Moreover, in that study, NPAS4 was also detected in microsomal and synaptosomal fractions (20). These findings suggest that NPAS4 contains motifs that direct its localization to different cellular compartments.

In our present study, we sought to perform a detailed characterization of the subcellular localization motifs of NPAS4 protein. We found that 24 h after transfection, NPAS4 was exchanged between the nucleus the and cytoplasm in COS-7 and Neuro 2a (N2a) cells. However, 48 h after transfection, we observed a dominantly nuclear distribution of NPAS4, especially under high-glucose medium conditions. We showed that NPAS4 contains NLSs and NESs in different regions of its protein structure. Interestingly, the NLSs and NESs of NPAS4 appear as partially overlapping pairs or in close proximity. This finding raises the possibility of alternating the activity of these signals and suggests that the interaction of the NLS of NPAS4 with importin prevents the interaction of the NES of NPAS4 with exportin and vice versa. This dynamic may play a role in the regulation of NPAS4 cellular shuttling. We suggest glucose concentration as one of factors that putatively influences the transport of NPAS4 protein. Additionally, in this study, experiments with LMB revealed that the activities of NESs are exportin-dependent.

## Results

### Subcellular localization of NPAS4 in COS-7 and Neuro 2a cells

**Figure 1.**
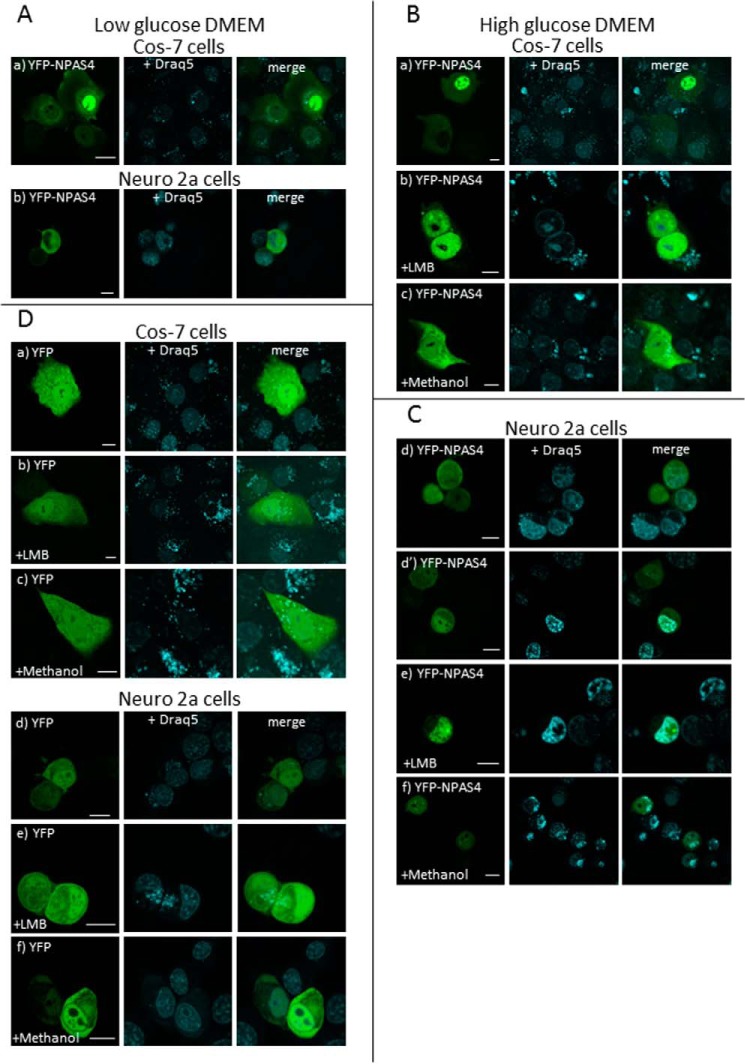
**Subcellular distribution of full-length NPAS4.** Expression of YFP-tagged full-length NPAS4 revealed distinct localization of fluorescence. Subcellular localization of expressed proteins in COS-7 and N2a cells was analyzed by confocal microscopy 20–24 h after transfection. Nuclei and nucleoli were stained by Draq5. *Bar*, 10 μm. *A*, representative images (single confocal plane) of the subcellular distribution of YFP-NPAS4 in COS-7 cells (*a*) and N2a cells (*b*) in low-glucose medium. *B*, representative images (single confocal plane) of the subcellular distribution of YFP-NPAS4 in cells cultured in high-glucose medium. Distribution of YFP-NPAS4 in COS-7 cells is shown under normal conditions (*a*), after LMB addition (*b*), and after methanol addition (*c*). *C*, distribution YFP-NPAS4 in N2a cells under normal conditions (*a* and *a*′), after LMB addition (*b*), and after methanol addition (*c*). *D*, representative images (single confocal plane) of the subcellular distribution of YFP-NPAS4 in cells cultured in high-glucose medium. Distribution in COS-7 cells is depicted under normal conditions (*a*), after LMB addition (*b*), and after methanol addition (*c*). Distribution in N2a cells is depicted under normal conditions (*d*), after LMB addition (*e*), and after methanol addition (*f*).

Chalfie *et al.* (22) describe that green fluorescent protein (GFP) can be used to monitor protein expression and localization in living organisms. Labeling proteins with different fluorescent proteins is currently a widely popular method that not only affects the localization but also the function of fused protein (23). Twenty-four hours after transfecting COS-7 and N2a cells, we analyzed the subcellular localization of yellow fluorescent protein (YFP)–tagged full-length NPAS4 and its derivatives by confocal and fluorescence microscopy. The expression of YFP-tagged NPAS4 and its derivatives in COS-7 cells was confirmed by Western blot analysis using an anti-GFP antibody (Fig. S1). In our experiments using low-glucose Dulbecco's modified Eagle's medium (DMEM), we noticed that the distribution of full-length NPAS4 in COS-7 and N2a cells 24 h after transfection was not exclusively nuclear. In particular, we observed more fluorescent protein in the cytoplasm (over 80% in COS-7 cells and 90% in N2a cells) than in the nucleus (less than 20% in COS-7 cells and 10% in N2a cells) (Figs. 1*A*, *a* and *b*, and S2*A*). Repeating the experiments under high-glucose conditions, the proportion of cells with nuclear localization of NPAS4 to cells with cytoplasmic localization of NPAS4 increased to 80/20 among COS-7 cells (Fig. 1*B*, *a*) and 30/70 among N2a cells 24 after transfection (Figs. 1*C*, *a* and *a′*, and S2*A*). Forty-eight hours after transfection, this proportion reached 90/10 in COS-7 cells and 50/50 in N2a cells (Fig. S2*A*). Expression of YFP resulted in ubiquitous localization in analyzed COS-7 cells independent of glucose concentration (Figs. 1*D* and S2*B*). Our results suggested that NPAS4 possess motifs that function as NLSs and NESs. First, we used LMB, a known classical NES-dependent transport inhibitor (17–19) to verify our hypothesis regarding the presence of an NES in NPAS4. Because we observed a decreasing number of live cells expressing YFP-NPAS4 after 48 h, we decided to perform further imaging experiments 24 h after transfection. Additional experiments in N2a cells were performed only in high-glucose medium, whereas additional experiments in COS-7 cells were performed in both high- and low-glucose medium. The addition of LMB to COS-7 and N2a cells resulted in strictly nuclear localization of NPAS4 (Figs. 1*B*, *b*, 1*C*, *b*, and S3*A*), indicating the inhibition of protein shuttling between the nucleus and the cytoplasm. To prove that methanol (LMB solvent) is not responsible for changes in protein localization after LMB addition, we used methanol alone, and we observed variable localization of NPAS4 similar to the results without LMB addition (Figs. 1*B*, *c*, 1*C*, *c*, and S3*A*). Interestingly, we did not observe a simultaneous localization of full-length NPAS4 in both the cell nucleus and cytoplasm. Retention of NPAS4 in the nucleus after LMB addition suggested the presence of an active NLS in this protein. To confirm that the influence of LMB on NPAS4 subcellular localization was specific, we expressed YFP alone in COS-7 and N2a cells and observed a ubiquitous distribution of YFP fluorescence that was independent of the presence or absence of LMB or methanol (Figs. 1*D* and S3*A*). Based on our results, we hypothesized that NPAS4 contains a sequence (or several sequences) that is responsible for its translocation to the nucleus (NLS) and a sequence (or several sequences) responsible for its translocation to the cytoplasm.

**Figure 2.**
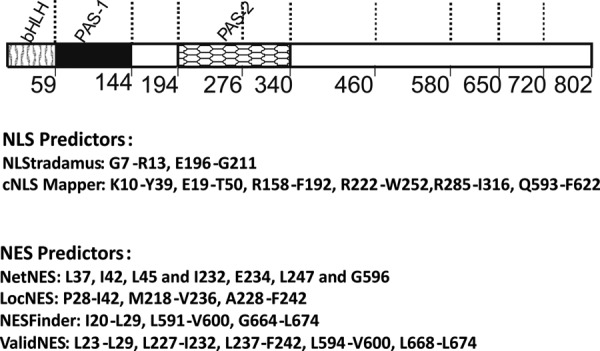
**NPAS4 schematic representation and NLS/NES prediction.** *Top*, schematic representation of NPAS4 protein. Regions of NPAS4 are depicted using different patterns. The length of each domain in the diagram is arbitrary. *Middle*, results of NLS predictors, with NPAS4 amino acid residues predicted as part of NLSs. *Bottom*, results of NES predictors, with NPAS4 amino acid residues predicted as part of NESs.

To test this hypothesis, we performed a set of *in silico* analyses of the NPAS4 sequence using various NLS and NES predictors. Detailed results of predictions are presented in Fig. 2. NucPred and SeqNLS did not predict any putative NLS sequence. NLStradamus, using a cut-off value of 0.1, predicted two potential NLS sequences encompassing amino acid (aa) residues Gly^7^–Arg^13^ in the bHLH domain and Glu^196^–Gly^211^ in the PAS-2 domain (Fig. 2, *top* and *middle*). When using a low cut-off value, cNLS Mapper identified five putative bipartite NLSs located in the following different regions of NPAS4: Lys^10^–Tyr^39^ and Glu^19^–Thr^50^ (bHLH domain), Arg^158^–Phe^192^ (region adjacent to PAS-2), Arg^222^–Trp^252^ (PAS-2), Arg^285^—Ile^316^ (PAC), and Gln^593^–Phe^622^ (C-terminal region) (Fig. 2, *top* and *middle*). NetNES predicted residues Leu^37^, Ile^42^, and Leu^45^ as part of a putative NES (bHLH domain); Ile^232^, Glu^234^, and Leu^247^ as a second NES (PAS-2); and Gly^596^ as part of a third NES (C-terminal region) (Fig. 2, *top* and *bottom*). LocNES predicted residues Pro^28^–Ile^42^ as the first NES (bHLH) and Met^218^–Val^236^ or Ala^228^–Phe^242^ as the second NES (PAS-2) (Fig. 2, *top* and *bottom*). NESFinder predicted residues Ile^20^–Leu^29^ (bHLH domain) and Leu^591^–Leu^604^ and Gly^664^–Leu^674^ (C-terminal region) (Fig. 2, *top* and *bottom*). ValidNES predicted putative NES residues Leu^23^–Leu^29^ (bHLH domain), Leu^227^–Ile^232^ or Leu^237^–Phe^242^ (PAS-2), and Leu^594^–Val^600^ and Leu^668^–Leu^674^ (C-terminal region) (Fig. 2, *top* and *bottom*). However, the results obtained by the different predictors were not fully consistent.

Nevertheless, the *in silico* analysis suggested the presence of NLSs in the bHLH domain, the PAS-2 domain, and the C-terminal region of NPAS4. Accordingly, putative NESs were expected to be located in the bHLH domain, the PAS-2 domain, and the C-terminal region of NPAS4. Interestingly, the predicted NLS and NES motifs seemed to be located within a very close distance, and thus we next verified these results experimentally.

### The bHLH domain of NPAS4 possesses both NLS and NES motifs

**Figure 3.**
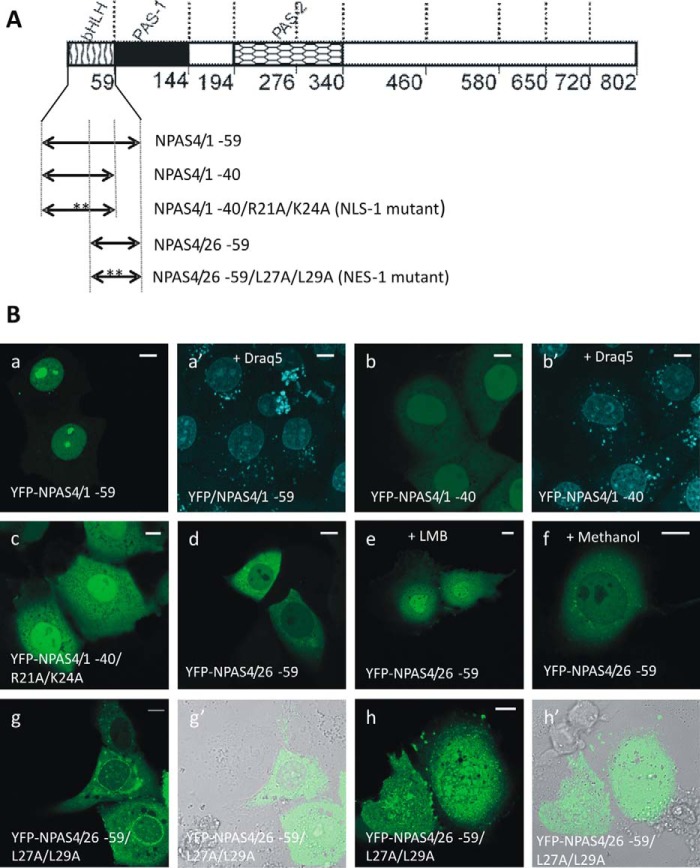
**Subcellular distribution of the bHLH domain of NPAS4 and their derivatives in COS-7 cells in low-glucose medium.** Subcellular localizations of the expressed YFP-tagged proteins were analyzed by confocal microscopy 20–24 h after transfection. *A*, schematic representation of NPAS4 protein and YFP-tagged derivatives of the bHLH domain. Regions of NPAS4 are depicted using different patterns. The length of each domain in the diagram is arbitrary. *B*, representative images (single confocal plane) of typical (presented by more than 95% of cells unless stated otherwise) subcellular distributions of the YFP-tagged derivatives of the NPAS4 bHLH domain in low-glucose medium are presented. Nuclei and nucleoli were stained with Draq5. *Bar*, 10 μm. Shown are YFP-NPAS4/1–59 (*a* and *a*′), YFP-NPAS4/1–40 (*b* and *b*′), YFP-NPAS4/1–40/R21A/K24A (*c*), and YFP-NPAS4/26–59 (*d*) under normal conditions; YFP-NPAS4/26–59 after LMB addition (*e*); YFP-NPAS4/26–59 after methanol addition (*f*); and prevailing patterns of YFP-NPAS4/26–59/L27A/L29A distributions (*g* and *g*′, *h* and *h*′).

To identify the putative NLS and NES motifs in the bHLH domain, we generated N-terminally YFP-tagged derivatives of the NPAS4 bHLH domain. To ensure that we did not disrupt structural motifs within the NPAS4 sequence, truncation mutants were designed according to the domain architecture (1, 2, 24) and putative secondary structure motifs of this protein. We used Draq5 DNA dye to visualize the nuclei and nucleoli. In contrast to full-length NPAS4, the YFP-labeled bHLH domain (Fig. 3*A*, YFP-NPAS4/1–59) presented a nuclear and nucleolar distribution in COS-7 cells in low-glucose DMEM (Fig. 3*B*, *a* and *a*′) and in N2a cells in high-glucose DMEM (Fig. S4). Interestingly, expression of the bHLH domain in COS-7 cells in high-glucose conditions resulted in the appearance of few (<5%) cells presenting both cytoplasmic and nucleolar fluorescence (Fig. S4). We performed an *in silico* prediction of the nucleolar localization signal (NoLS) in NPAS4 with the nucleolar localization sequence detector (NoD) and found that the bHLH domain was negative for any NoLS (score of 0.75). However, previously performed *in silico* predictions of NLSs and NESs in NPAS4 showed that a partially overlapping NLS and NES could be present in this region (Fig. 2). To confirm these overlapping signals experimentally, we prepared constructs including aa 1–40 and 26–59 (Fig. 3*A*). Deletion of aa 41–59 resulted in a weakening of the fluorescence signal of YFP-NPAS4/1–40 in the nucleoli. Additionally, although this protein fragment could still enter the nucleus, it also appeared in the cytoplasm of COS-7 and N2a cells, indicating weak NLS signal activity, probably due to the absence of the basic amino acids Arg^51^ and Lys^52^ (Figs. 3*B*, *b* and *b*′, and S5). As NLS activity typically depends on the presence of basic amino acid residues (16, 25), we prepared a mutant including a 1–40–aa fragment, where Arg^21^ and Lys^24^ were substituted by Ala (Fig. 3*A*). The localization of this mutant shifted from predominantly nuclear for the unmodified fragment (Figs. 3*B*, *b*, and S5) to only slightly predominantly nuclear. Additionally, this mutant was unequivocally excluded from the nucleoli (Figs. 3*B*, *c*, and S5), presenting reasonably low NLS activity and no NoLS activity in the nucleoli. Based on the results from cNLS Mapper (Fig. 2*B*) and our experiments, we concluded that the bHLH domain contains multipartite NLS motifs comprising aa 10–52 (^10^**K**A**RR**DQINAEI**R**NL**K**ELLPLAEAD**K**V**R**LSYLHIMSLACIYT**RK**^52^, where bold are basic residues and bold and underlined are basic substituted residues). Additionally, this sequence was found to be linked directly to the NoLS, in which aa ^51^RK^52^ are indispensable for NoLS activity.

The YFP-NPAS4/26–59 truncation mutant was predominantly cytoplasmic (Figs. 3*B*, *d*, and S5) because of the predicted NES. We then used LMB to verify the presence of NES in this fragment. Although we observed a clear shift in localization from cytoplasmic to predominantly nuclear (Fig. 3*B*, *e*), the addition of methanol as a control did not influence localization (Fig. 3*B*, *f*). As classical NES activity depends on the presence of hydrophobic residues (usually Leu) (25), we next substituted Leu^27^ and Leu^29^ with Ala in the 26–50–aa bHLH fragment tagged with YFP (Fig. 3*A*, YFP-NPAS4/26–50/L27A/L29A). Expression of this point mutant resulted in fluorescence in both the nucleus and cytoplasm as opposed to the diffuse cytoplasmic fluorescence of the unmodified truncated mutant. Interestingly, although protein aggregates appeared around the nuclear membrane in some cells (Fig. 3*B*, *g* and *g*′), these aggregates in other cells were visible throughout the whole cell (Fig. 3*B*, *h* and *h*′). This experiment proved that residues Leu^27^ and Leu^29^ are crucial for leucine-rich NES activity in the bHLH domain. Additionally, dominant nuclear localization of the YFP-NPAS4/1–40 mutant (Fig. 3*B*, *b*) lacking Leu^45^ indicated the importance of this amino acid residue for NES activity. Based on results from the NetNES and LocNES predictors (Fig. 2*C*), we concluded that the bHLH domain contains the sequence ^26^**LL**P**L**AEADKVR**L**SY**L**HIMS**L**^45^ (where bold are hydrophobic residues and bold and underlined are hydrophobic substituted residues) and exhibits NES activity.

Altogether, our results show that the bHLH domain contains two overlapping sequences that are active NLS and NES motifs. Additionally, nuclear and nucleolar localization motifs of the bHLH domain of NPAS4 are directly linked.

### Detection of cellular localization signals in the PAS domains of NPAS4

To identify putative NLS and NES motifs in the PAS-1 and PAS-2 domains, we generated the respective N-terminally YFP-tagged derivatives of the PAS domains of NPAS4 and analyzed their subcellular localization in COS-7 and N2a cells using fluorescence microscopy. For visualization of nuclei and nucleoli, we used Draq5 DNA dye.

**Figure 4.**
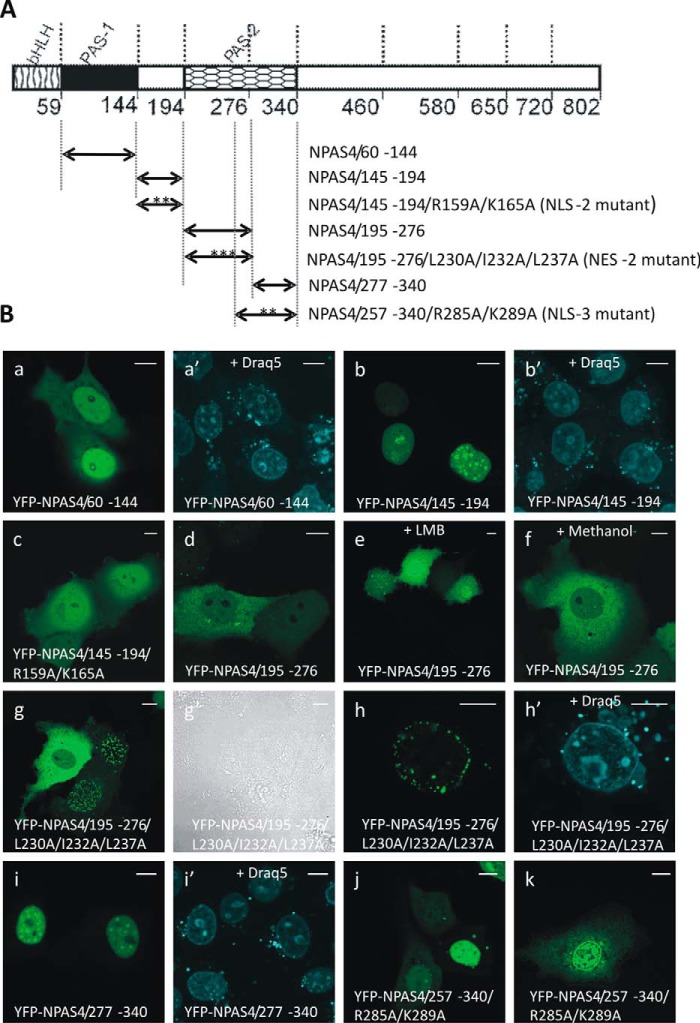
**Subcellular distributions of the PAS domains of NPAS4 and their derivatives in COS-7 cells in low-glucose medium.** Subcellular localizations of the expressed YFP-tagged proteins were analyzed by confocal microscopy 20–24 h after transfection. *A*, schematic representation of NPAS4 protein and YFP-tagged derivatives of the PAS domains. Regions of NPAS4 are depicted using different patterns. The length of each domain in the diagram is arbitrary. *B*, representative images (single confocal plane) of typical (presented by more than 95% of cells unless stated otherwise) subcellular distributions of the YFP-tagged derivatives of the NPAS4 PAS domains in low-glucose medium are presented. Nuclei and nucleoli were stained with Draq5. *Bar*, 10 μm. Shown are YFP-NPAS4/60–144 (*a* and *a*′), YFP-NPAS4/145–194 (*b* and *b*′), YFP-NPAS4/145–194//R159A/K165A (*c*), and YFP-NPAS4/195–276 (*d*) under normal conditions; YFP-NPAS4/195–276 after LMB addition (*e*); YFP-NPAS4/195–276 after methanol addition (*f*); prevailing patterns of YFP-NPAS4/195–276/L230A/L232A/L237A distributions (*g*, *g*′, *h*, and *h*′); YFP-NPAS4/277–340 (*i* and *i*′); prevailing patterns of YFP-NPAS4/257–340/R285A/K289A distribution (*j* and *k*).

We found that the YFP-NPAS4/60–144 fragment containing the PAS-1 domain (Fig. 4*A*) expressed in COS-7 cells in low-glucose medium was localized predominantly to the cell nucleus (Fig. 4*B*, *a* and *a*′), although no putative NLS was detected by known NLS predictors (see Fig. 2). Additionally, the only basic amino acid residues (characteristic of classical NLS) present in this region were Lys^93^ and Arg^132^. Expression of this fragment both in COS-7 and N2a cells in high-glucose DMEM resulted in ubiquitous distribution (Fig. S4).

The fragment linking the PAS-1 and PAS-2 domains, encompassing aa 145 to 194 (Fig. 4*A*), was localized exclusively to the nucleus of all analyzed COS-7 and N2a cells independent of glucose concentration (Figs. 4*B*, *b* and *b*′, and S4), indicating the presence of NLS activity. In some cells, fluorescence was also observed in the nucleoli; however, this localization was not present in all cells and was not as obvious as the localization of the bHLH domain mutants (data not shown). Additionally, the NoLS prediction results were negative. The cNLS Mapper predictor identified the sequence ^158^RRQSAGNKLVLIRGRFHAHPPGAYWAGNPVFTAF^191^ as a putative NLS (Fig. 2*B*). To validate this prediction, we generated a YFP-NPAS4/145–194 construct with R159A and K165A substitutions (Fig. 4*A*). In contrast to the WT fragment, the YFP-NPAS4/145–194/R159A/K165A mutant was localized to both compartments of analyzed cells (Fig. 4*B*, *c*), thus confirming the switch to an active NLS in this region.

To identify the localization of signals in the PAS-2 domain of NPAS4, we generated a YFP-NPAS4/195–276 construct, which is an NPAS4 fragment containing the N-terminal part of the PAS-2 domain (Fig. 4*A*). This fragment mainly exhibited a cytoplasmic distribution both in COS-7 cells in low-glucose medium (Fig. 4*B*, *d*) and in COS-7 and N2a cells in high-glucose medium, indicating that the presence of NES activity was consistent with the NES predictions (Fig. 2*C*). To verify the hypothesis regarding the presence of an active NES motif within the PAS-2 domain, we analyzed this YFP-tagged fragment using LMB. The addition of LMB shifted the localization of the YFP-NPAS4/195–276 fragment from cytoplasmic to slightly predominantly nuclear (Fig. 4*B*, *e*), confirming the inhibition of the NES-exportin 1 interaction. Furthermore, the addition of methanol as an LMB solvent did not change the cytoplasmic localization of YFP-NPAS4/195–276 (Fig. 4*B*, *f*), which confirmed its lack of influence on NES activity. Analysis of the NES prediction results led us to hypothesize that hydrophobic residues Leu^230^, Ile^232^, and Leu^237^ may be part of the putative NES motif. To verify this hypothesis, we substituted these residues with Ala (Fig. 4*A*). Surprisingly, the resulting protein, YFP-NPAS4/195–276/L23A/I232A/L237A, seemed to localize around the nuclear membrane of most of the analyzed cells (Fig. 4*B*, *g*). To clarify the precise localization of the YFP-NPAS4/195–276/L230/I232/L237 mutant, we compared fluorescence and transmitted light images of the same cells (Fig. 4*B*, *g*′). However, we could not definitively determine whether the expressed fluorescent aggregates were visible inside or outside the nuclear membrane or if they were integrated into the nuclear membrane (Fig. 4*B*, *h*), despite using Draq5 DNA dye to visualize cell nuclei (Fig. 4*B*, *h*′). Analysis of the NPAS4/195–276 fragment using NLS predictors was not consistent; whereas NLStradamus predicted Glu^196^–Gly^211^ as a putative NLS, cNLS Mapper predicted Arg^222^–Trp^252^ as a putative NLS (Fig. 2*B*). Next, we generated a YFP-tagged fragment comprising aa 277–340; this fragment consisted of the C-terminal part of the PAS-2 domain, which is also known as the PAS domain-associated C terminus (PAC) (Fig. 4*A*). The YFP-NPAS4/277–340 fragment was found to localize exclusively to the nucleus of all analyzed COS-7 and N2a cells (Figs. 4*B*, *i* and *i*′, and S4), which is consistent with the cNLS Mapper prediction of the ^285^RLQAKHGGWTWIYCMLYSDGPEGPITANNYPI^316^ sequence as a putative NLS (Fig. 2*B*). To confirm the presence of an active NLS, we constructed a point mutant with R285A and K289A substitutions (Fig. 4*A*). However, the results of YFP-NPAS4/257–340/R285A/K289A mutant expression were ambiguous. Three different distribution patterns were observed, and each pattern was found in a similar number of cells (cells with an equal distribution in both nuclear and cytoplasmic compartments, cells with a slightly predominantly nuclear distribution, and cells with a predominantly nuclear distribution (Fig. 4*B*, *j*)), which was in contrast to the exclusively nuclear localization of the YFP-NPAS4/277–340 fragment without substitutions (see Fig. 4*B*, *i*). Interestingly, in cells presenting dominant nuclear localization of YFP-NPAS4/257–340/R285A/K289A, we observed a change in the distribution pattern in the nucleoplasm (Fig. 4*B*, *k*).

Our results showed that in the region adjacent to the PAS-2 domain and within this domain, NLS and NES sequences are located in close proximity: NLS, ^158^**R****R**QSAGN**K**LVLI**R**G**R**FHAHPPGAYWAGNPVFTAF^191^ (where bold are basic residues, and bold and underlined are basic substituted residues); NES, ^227^**L**A**L****L**D**I**SESV**L**ITY**L**GF-242 (where bold are hydrophobic residues and bold and underlined are hydrophobic substituted residues); and putative NLS, ^285^**R**LQA**K**H GGWTWIYCMLYSDGPEGPITANNYPI^316^ (where bold and underlined are basic substituted residues). Thus, in addition to the bHLH domain, the PAS domains also seem to play an important role in the final NPAS4 localization.

### Detection of localization signals in the C-terminal NPAS4 fragment

**Figure 5.**
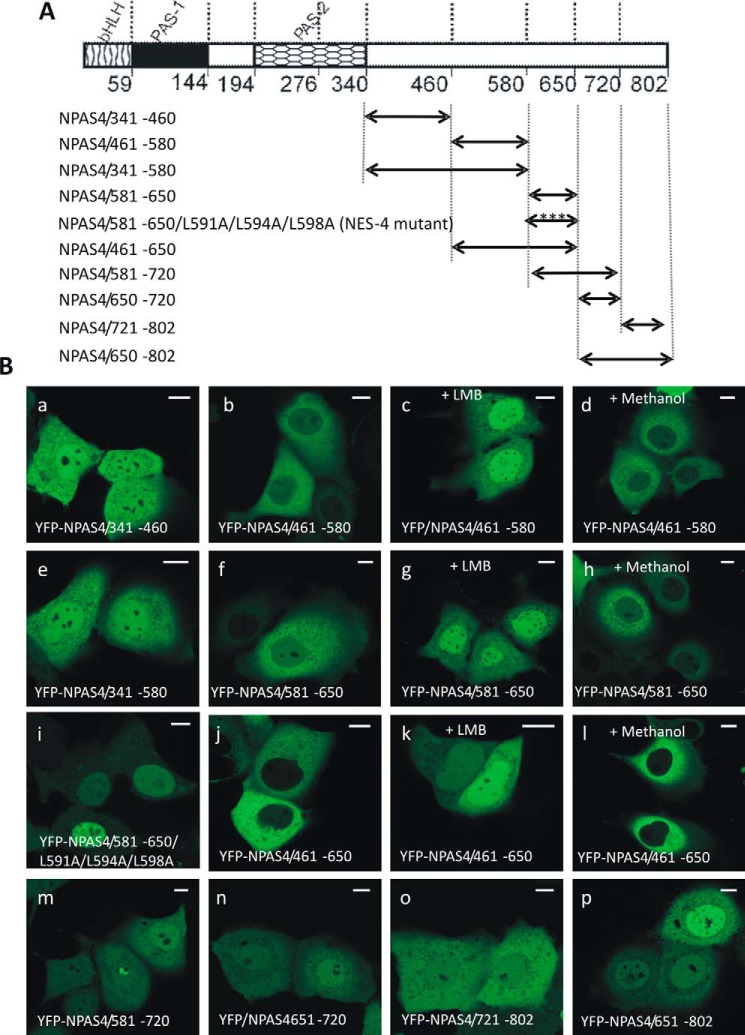
**Subcellular distribution of the C-terminal part of NPAS4 derivatives in COS-7 cells in low-glucose medium.** Subcellular localizations of the expressed YFP-tagged proteins were analyzed by confocal microscopy 20–24 h after transfecting COS-7 cells in low-glucose medium. *A*, schematic representation of NPAS4 protein and YFP-tagged derivatives of NPAS4 C terminus. Regions of NPAS4 are depicted using different patterns. The length of each domain in the diagram is arbitrary. *B*, representative images (single confocal plane) of typical (presented by more than 95% of cells) subcellular distributions of the YFP-tagged derivatives of the NPAS4 C terminus are presented. *Bar*, 10 μm. Shown are YFP-NPAS4/341–460 (*a*) and YFP-NPAS4/461–580 (*b*) under normal conditions; YFP-NPAS4/461–580 after LMB addition (*c*); YFP-NPAS4/461–580 after methanol addition (*d*); YFP-NPAS4/341–580 (*e*) and YFP-NPAS4/581–650 under normal conditions (*f*); YFP-NPAS4/581–650 after LMB addition (*g*); YFP-NPAS4/581–650 after methanol addition (*h*); YFP-NPAS4/581–650/L591A/L594A/L598A (*i*) and YFP-NPAS4/461–650 under normal conditions (*j*); YFP-NPAS4/461–650 after LBM addition (*k*); YFP-NPAS4/461–650 after methanol addition (*l*); YFP-NPAS4/581–720 (*m*); YFP-NPAS4/651–720 (*n*); YFP-NPAS4/721–802 (*o*); and YFP-NPAS4/651–802 (*p*).

To test precisely for the presence of subcellular localization signals in the C-terminal region of NPAS4 encompassing residues 341 to 802, we analyzed fragments of this region separately. These NPAS4 fragments were defined according to the secondary structure prediction, which was performed using PSIPRED. Eventually, five fragments encompassing residues 341–460, 461–580, 581–650, 650–720, and 721–802 were selected. YFP-tagged NPAS4/341–460 (Fig. 5*A*) was distributed ubiquitously throughout the whole cell, both in COS-7 cells independent of glucose concentration and in N2a cells (Figs. 5*B*, *a*, and S6). As no NLS or NES motif was predicted in this area, this result proved the lack of subcellular localization signals in this region. Surprisingly, the next fragment, YFP-NPAS4/461–580 (Fig. 5*A*), localized to the cytoplasm both in COS-7 cells independent of glucose condition and in N2a cells (Figs. 5*B*, *b*, and S6). The addition of LMB shifted the localization of YFP-NPAS4/461–580 to the nucleus (Fig. 5*B*, *c*), thus proving the presence of an active NES motif interacting with exportin 1. Methanol, which was used as a control, did not influence localization (Fig. 5*B*, *d*). Interestingly, none of the NES predictors suggested the presence of a putative NES motif in this fragment. We decided to investigate the effect of truncation by creating a YFP-NPAS4/341–580 construct (Fig. 5*A*) including a fragment with cytoplasmic localization (YFP-NPAS4/461–580) and a fragment with ubiquitous localization (YFP-NPAS4/341–460). Expression of this combined YFP-NPAS4/341–580 fragment, containing both previously tested fragments, resulted in fluorescence in both cellular compartments (cytoplasm and nucleus) (Figs. 5*B*, *e*, and S7). We hypothesized that some residues in the 341–460–aa fragment deactivate the putative NES in the 461–580–aa fragment by interacting with an unknown factor, thus masking the NES. As a typical NES sequence is rich in Leu residues, we performed additional analyses of the sequence of this region (aa 461–580). However, we did not find any obvious cluster of Leu residues, and we were unable to identify the sequence possessing NES activity in this fragment. The localization of YFP-NPAS4/581–650 (Fig. 5*A*) was cytoplasmic in all analyzed cells, both in low- and high-glucose medium (Figs. 5*B*, *f*, and S6); this result is in full agreement with most NES predictors identifying amino acid residues between Leu^591^ and Leu^604^ as a putative NES motif (see Fig. 2). Moreover, the addition of LMB shifted the cytoplasmic fluorescence of this mutant to a predominantly nuclear localization (Fig. 5*B*, *g*). Methanol addition did not change the localization of the fluorescence signal (Fig. 5*B*, *h*). Next, we examined this fragment by substituting amino acid residues suspected to be part of the hypothesized NES motif. We substituted Leu^591^, Leu^594^, and Leu^598^ with Ala (Fig. 5*A*) and observed a clear shift in fluorescence to the nucleoplasm upon expression of the YFP-NPAS4/581–650/L591A/L594A/L598A mutant (Fig. 5*B*, *i*), indicating deactivation of NES activity. This result confirmed that the predicted sequence, ^591^LAQLRGPLSV^600^, is an active NES. To better explain the nuclear localization of this mutant, we further analyzed results from the NLS predictions. cNLS Mapper predicted the sequence ^593^QLRGPLSVDVPLVPEGLLTPEASPVKQSFF^622^ as a putative NLS (Fig. 2). This sequence partially overlapped with a region that was previously identified to contain an NES (591–600 aa) but seemed to act predominantly as an NLS. As we were interested in the influence of additional amino acid residues on NLS and NES activities, we generated YFP-tagged combined fragments YFP-NPAS4/461–650 and YFP-NPAS4/581–720 (Fig. 5*A*) containing both detected signals. Expression of YFP-NPAS4/461–650 resulted in exclusive cytoplasmic fluorescence in COS-7 cells both in low- and high-glucose conditions and in N2a cells (Figs. 5*B*, *j*, and S7), thus predominantly presenting characteristics of the NES in the tested region. The addition of LMB resulted in the uniform localization of the mutant in both compartments of COS-7 cells in low-glucose medium and in N2a cells (Figs. 5*B*, *k*, and S3*B*); however, in high-glucose DMEM, we observed an evident shift in localization of the expression of this fragment to the nucleus after LMB addition (Fig. S3*B*). We expected exclusive nuclear localization of YFP-461–650 after LMB addition because the Leu^594^–Phe^622^ sequence present in this fragment was a predicted NLS and was putatively responsible for the nuclear localization of the YFP-NPAS4/581–650/L591A/L594A/L598A mutant. Therefore, we have no clear explanation for the different localization behavior observed in COS-7 cells depending on glucose concentration. One possibility is that the presence of additional amino acid residues (aa 461–580) results in NLS activity inhibition in low-glucose medium via an unknown mechanism. The methanol control did not influence localization (Fig. 5*B*, *l*). Expression of the YFP-NPAS4/581–720 fragment (Fig. 5*A*) resulted in ubiquitous fluorescence in both the nucleus and the cytoplasm of the analyzed cells (Figs. 5*B*, *m*, and S7). Thus, in this mutant, the *strength* of the NLS and NES activity seems to be balanced. Next, we expressed NPAS4 fragments YFP-NPAS4/651–720 and YFP-NPAS4/721–802 (Fig. 5*A*), and these fragments localized to both the nucleus and the cytoplasm (Figs. 5*B*, *n* and *o*, and S6). We then fused these fragments and found that the combined YFP-NPAS4/651–802 fragment (Fig. 5*A*) localized to both cellular compartments, with a slightly more robust fluorescent signal in the nucleus (Figs. 5*B*, *p*, and S7). This result is inconsistent with the prediction results, such that ValidNES identified the Leu^668^–Leu^674^ sequence and NESFinder identified the Gly^664^–Leu^674^ sequence as putative NES motifs (see Fig. 2). Therefore, these results verified that this sequence is not an active NES and that its activity is inhibited by an unknown factor.

Altogether, our results show that the C-terminal region of NPAS4 contains two overlapping sequences: ^591^**L**AQ**L**RGP**L**SV^600^ (bold and underlined are hydrophobic substituted residues) is an active NES and ^593^QL**R**GPLSVDVPLVPEGLLTPEASPVKQSFF^622^ (bold is the only basic residue) is a putative NLS. Additionally, we detected NES activity in the 460–580–aa region of the C-terminal part of NPAS4, but we were unable to identify the sequence responsible for directing this fragment to the cytoplasm. Thus, we believe that in addition to the bHLH and PAS domains, the C terminus of NPAS4 contributes to the regulation of NPAS4 shuttling in response to different stimuli.

### The hierarchy of the cellular localization signals in NPAS4

**Figure 6.**
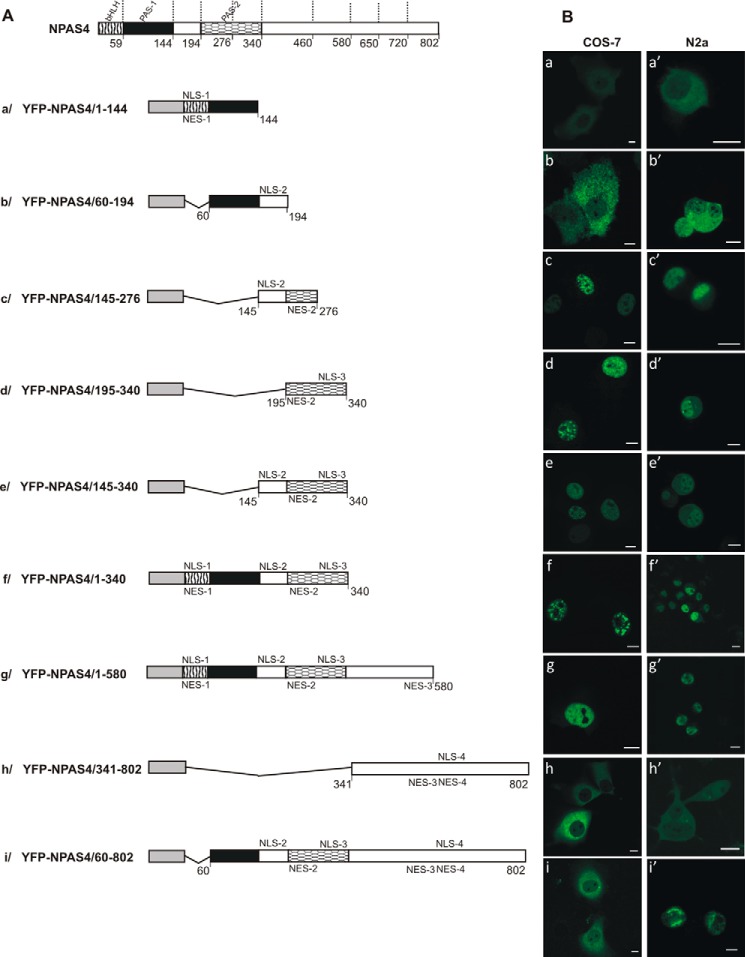
**Hierarchy of NLS and NES signals in NPAS4.** Subcellular localizations of the expressed YFP-tagged derivatives of NPAS4 were analyzed by confocal microscopy 20–24 h after transfecting COS-7 and N2a cells. *A*, schematic presentation of NPAS4 and YFP-tagged NPAS4 derivatives. *B*, subcellular distributions of YFP-tagged derivatives of NPAS4. Representative images (single confocal plane) of typical (presented by more than 95% of cells unless stated otherwise) subcellular distributions of the NPAS4 derivatives are presented. Nuclei and nucleoli were stained by Draq5. *Bar*, 10 μm. Distributions in COS-7 cells in low-glucose medium: YFP-NPAS4/1–144 (*a*), YFP-NPAS4/60–144 (*b*), YFP-NPAS4/145–276 (*c*), YFP-NPAS4/195–340 (*d*), YFP-NPAS4/145–340 (*e*), YFP-NPAS4/1–340 (*f*), YFP-NPAS4/1–580 (*g*), YFP-NPAS4/341–802 (*h*), and YFP-NPAS4/60–802 (*i*). Distributions in N2a cells in high-glucose medium: YFP-NPAS4/1–144 (*a*′), YFP-NPAS4/60–144 (*b*′), YFP-NPAS4/145–276 (*c*′), YFP-NPAS4/195–340 (*d*′), YFP-NPAS4/145–340 (*e*′), YFP-NPAS4/1–340 (*f*′), YFP-NPAS4/1–580 (*g*′), YFP-NPAS4/341–802 (*h*′), and YFP-NPAS4/60–802 (*i*′).

As we detected more than one NLS and one NES in NPAS4, we next investigated the interplay of NPAS4 localization signals. We generated YFP-tagged derivatives of NPAS4 containing different domains and analyzed their subcellular localization in COS-7 and N2a cells. The expression of YFP-tagged derivatives in COS-7 cells was confirmed by Western blot analysis using an anti-GFP antibody (see Fig. S1). To visualize the nuclei and nucleoli, we used Draq5 DNA dye. First, we sought to verify the hierarchy of signals in the N-terminal part of NPAS4. By tagging with YFP, we prepared fragments containing the bHLH and PAS-1 domains (Fig. 6*A*, *a*, YFP-NPAS4/1–144); the PAS-1 domain and a linker between the PAS-1 and PAS-2 domains (Fig. 6*A*, *b*, YFP-NPAS4/60–195); a linker between the PAS-1 and PAS-2 domains connected to the N-terminal part of the PAS-2 domain (Fig. 6*A*, *c*, YFP-NPAS4/145–276); the PAS-2 domain (Fig. 6*A*, *d*, YFP-NPAS4/195–340); a linker between the PAS-1 and PAS-2 domains combined with the PAS-2 domain (Fig. 6*A*, *e*, YFP-NPAS4/145–340); and the N-terminal part of NPAS4 containing the bHLH, PAS-1 and PAS-2 domains with a linker (Fig. 6*A*, *f*, YFP-NPAS4/1–340).

The results of the expression of YFP-NPAS4/1–144 (Fig. 6*A*, *a*) and YFP-NPAS4/60–195 (Fig. 6*A*, *b*) fragments could not be easily interpreted because they were not fully consistent with our expectations. We observed predominantly cytoplasmic localization (>90%) in COS-7 and N2a cells in both low- and high-glucose medium (Figs. 6*B*, *a*, *a*′, *b*, and *b*′, and S8 and S9). Interestingly, in the case of YFP-NPAS4/1–144, few cells (<10%) presented simultaneous cytoplasmic and nucleolar localization (Fig. S9). Nucleolar and nuclear localization of YFP-NPAS4/1–144 could be interpreted easily because NLS-1 linked with NoLS in the bHLH domain and was dominant over NES-1 in the YFP-NPAS4/1–59–expressing fragment (see Fig. 3*B*, *a*), similar to the nuclear localization of YFP-NPAS4/60–195 comprising NLS-2 in the linker between the PAS domains (see Fig. 4*B*, *b*). However, we cannot provide a clear explanation for the cytoplasmic localization of the 1–144–aa and 60–194–aa fragments. The PAS-1 domain (60–144 aa), which is responsible for the cytoplasmic shift for both of these fragments, did not present any subcellular localization signal activity in our previous experiments (see Fig. 4*B*, *a*).

Strictly nuclear localization of the expressed YFP-NPAS4/145–276 fragment containing NLS-2 and NES-2 (Fig. 6*A*, *c*) in COS-7 and N2a cells (Figs. 6*B*, *c* and *c*′, and S8 and S9) showed that NLS-2 is dominant over NES-2. Similarly, the expression of YFP-NPAS4/195–340 (PAS-2 domain) containing NES-2 and NLS-3 (Fig. 6*A*, *d*) led to the observation of fluorescence in the nuclei of both of the cell types tested (Figs. 6*B*, *d* and *d*′, and S8 and S9), pointing to the dominant character of NLS-3 over NES-2. The fusion of these fragments in the YFP-NPAS4/145–340 construct comprising NLS-2, NES-2, and NLS-3 (Fig. 6*A*, *e*) resulted in the expected nuclear localization of the expressed protein in COS-7 and N2a cells (Figs. 6*B*, *e* and *e*′, and S8 and S9), proving the dominant characters of both NLS-2 and NLS-3 over NES-2. These results were independent of the glucose concentration used. Nuclear localization of the expressed N-terminal part of NPAS4 (Fig. 6*A*, *f*, YFP-NPAS4/1–340) in COS-7 and N2a cells (Figs. 6*B*, *f* and *f*′, and S8 and S10*A*) revealed a consequent domination of NLSs (NLS-1, NLS-2, and NLS-3) in this part of the protein.

Next, we evaluated the subcellular distribution of the NPAS4/1–580 fragment, which was attached to the previous fragment by 341–580 aa, containing NES-3 (Fig. 6*A*, *g*). Expression in both low- and high-glucose medium resulted in dominant nuclear localization of the expressed protein in COS-7 and N2a cells (Figs. 6*B*, *g* and *g*′, and S8 and S10*A*), indicating the domination of NLSs (NLS-1, NLS-2, and NLS-3) from the N-terminal part of NPAS4 over NES-3.

To test the hierarchy of the signal in the C-terminal part of NPAS4, we prepared the YFP-NPAS4/341–802 construct containing NES-3, NLS-4, and NES-4 (Fig. 6*A*, *h*). Expression of this fragment resulted in cytoplasmic localization in COS-7 and N2a cells independent of the glucose concentration (Figs. 6*B*, *h* and *h*′, and S8 and S10*A*). This result is consistent with the cytoplasmic distribution observed previously for the expression of YFP-NPAS4/581–650 (see Fig. 5*B*, *f*) and indicated domination of NES-4 over NLS-4.

To verify the hierarchy of dominant NLSs from the N-terminal part of NPAS4 and dominant NESs from the C-terminal part of NPAS4, we created NPAS4 with deletion of the bHLH domain (YFP-NPAS4/60–802) (Fig. 6*A*, *i*). The expressed protein was localized strictly in the cytoplasm of N2a cells in both low- and high-glucose medium (Figs. 6*B*, *i*′, and S8 and S10, *A* and *B*). Interestingly, expression of this construct in COS-7 cells resulted in cytoplasmic localization in the condition of low-glucose medium (Figs. 6*B*, *i*, and S8 and S10*B*), whereas high-glucose medium resulted in a localization shift to 80% cytoplasmic and 20% nuclear (Fig. S10*A*). This result proves that NESs from the C terminus of NPAS4 (definitively NES-4 and possibly NES-3) are dominant over both NLS-2 and NLS-3, which are dominant over NES-2. However, this domination may be dependent on the cell type and the glucose concentration used. As full-length NPAS4 localization is distributed between the nucleus and the cytoplasm depending on the glucose level in the medium and on the cell type (see Fig. 1), we suggest that the domination of NLS-1 from the bHLH domain over NESs from the C-terminal part of NPAS4 relies on various glucose level–dependent factors.

## Discussion

The eukaryotic cell is organized into membrane-covered compartments performing biochemically distinct cellular processes and possessing specific sets of proteins. Subcellular localization determines the access of proteins to interacting partners and the possibility of posttranslational modifications. The inappropriate localization of proteins has been linked to many human diseases, such as Alzheimer's disease, kidney stones, and cancer (26). As subcellular distribution is a key factor for the proper function of bHLH-PAS proteins (27), we performed a detailed characterization of the sequences responsible for NPAS4 protein transport between the cytoplasm and the nucleus.

**Figure 7.**
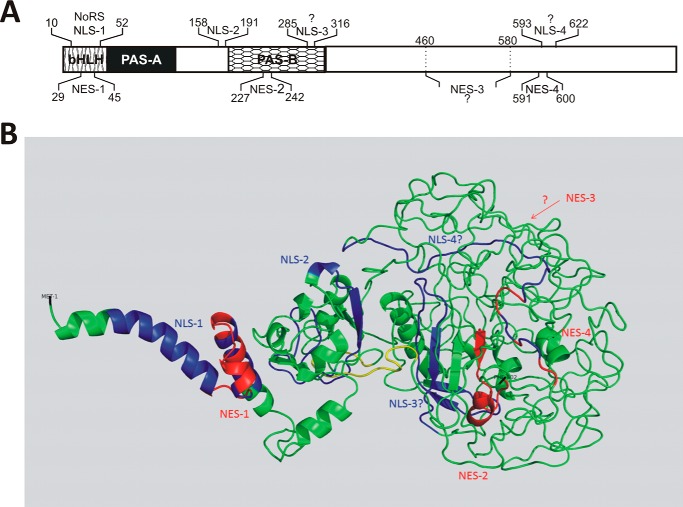
**Summary of NLSs and NESs in NPAS4.** *A*, schematic representation of NLSs and NESs residing within the NPAS4 protein structure. *B*, NLSs and NESs presented in a 3D model of NPAS4 generated by Phyre2 (59). Residues 1–340 encompassing the bHLH and PAS domains of NPAS4 were modeled with confidence higher than 90% using the templates c4f31B, c4zprB, C4f31A, c4zp4D, c5sy5B and c5sy7B. The C-terminal region of NPAS4 lacking any template and predicted as disordered was modeled *ab initio* (67% of the protein is predicted as disordered). NLS-1, NLS-2, and putative NLS-3 and NLS-4 are shown in *blue*. NES-1, NES-2, NES-3, and NES-4 are shown in *red*. The unique mammalian NPAS4 with sequence rich in PG and PR repeats, where the Pro^201^ residue was predicted (probability 0.85, HydroxyPred) as the site of hydroxylation (^197^PRPR**P**GPGPGPGPGP^211^), is shown in *yellow*.

We show here that the bHLH domain of NPAS4, responsible for ARNT binding and interaction with DNA, contains NLS-1 and overlapping NES-1 (Fig. 7*A*). Interestingly, our data indicate that NLS-1 is linked directly with NoLS. Based on our results, we hypothesized that the bHLH domain is an important player in the dynamic exchange of NPAS4 between subcellular compartments. Additionally, we documented the presence of NLS-2 and NES-2 sequences located in close proximity to the PAS-2 domain, which is usually responsible for ligand binding and sensing environmental cues. In the C-terminal region of the PAS-2 domain, also known as PAC, we hypothesize the presence of NLS-3. Until now, ligand binding by the PAS domains of NPAS4 has not been tested. In searching for an explanation for the biological significance of the presence of NLSs and NESs in PAS domains, we performed predictions of NPAS4 ligand binding using the 3DligandSite server. The results of our predictions suggest the possibility that the PAS-1 and PAS-2 domains bind to heme and flavin mononucleotide (FMN), which is consistent with the results of Wu *et al.* (5), who found that mammalian bHLH-PAS transcription factors bind multiple ligands. Furthermore, our hypothesis of an interaction with heme is substantiated by the fact that heme binding was documented for the PAS domains of another neuronal PAS domain–containing protein, NPAS2. Heme binding by NPAS2 forms a gas-regulated sensor; however, the natural ligand is currently unknown (28). FMN is present in light–oxygen–voltage (LOV) proteins, which belong to the PAS family, as a chromophore involved in blue-light absorption that is important, for example, in regulating circadian rhythms (29). The predicted ligand binding by the PAS-2 domain occurs partially via amino acids located in regions of detected NLS and NES sequences: NLS-2 (Thr^189^ and Phe^191^), NES-2 (Leu^227^), and NLS-3 (Arg^285^, Leu^286^, Ile^296^, Cys^298^, Leu^300^, Ile^309^, Thr^310^, Ala^311^, and Asn^313^); this suggests ligand binding–dependent regulation of NPAS4 subcellular localization. Interestingly, Western blot analysis of expressed WT PAC fused to YFP (YFP-NPAS4/277–340) resulted in the dual band (discussed later) migrating more slowly than expected (apparent molecular mass of the band of ∼45 kDa but an expected molecular mass of 34 kDa) (Fig. S1*B*). The substitution of Arg^285^ by alanine in the YFP-NPAS4/257–340/R285A/K289A mutant abolished this discrepancy, and Western blot analysis showed one band with an apparent molecular mass corresponding to the expected 36 kDa (Fig. S1*D*), which substantiates the hypothesis regarding the role of Arg^285^ in the ligand binding. Using the nine-amino-acid transactivation domain prediction tool (9aaTAD, data not shown), we found a perfect match for 9aaTAD encompassing aa 276–284, located next to NLS-3, which raised the possibility of connections among sensing the environment by ligand (*i.e.* heme) binding, the regulation of NLS-3 activity, and the interaction of 9aaTAD with the transcription machinery (30).

The presence of multiple opposing and partially overlapping localization signals suggests a complex mode of regulation of NPAS4 shuttling in response to different factors. NLS and NES sequences are short linear motifs. The preferential location of these motifs in the intrinsically disordered regions (IDR) of proteins has been found to enable flexible and easily accessible interactions with their binding partners (31). To substantiate our results, we performed predictions of NPAS4 disorder, and 67% of the NPAS4 sequence (mostly C-terminal) was predicted to be disordered (data not shown). As the structure of NPAS4 is unknown, we used Phyre2 to generate a molecular model of NPAS4 to predict the surface exposure of defined sequences. Localization signals can be structurally exposed and accessible to transport machinery or masked by interacting partners. The NLSs and NESs that we detected in the bHLH and PAS domains were located in or adjacent to short disordered regions, providing some degree of flexibility, despite their location in ordered domains. We believe that the location of NES-3, NES-4, and NLS-4 in the C-terminal, as a long IDR of NPAS4, serves to enhance flexibility and precise mechanistic control of NPAS4 activity in the context of interactions with its coactivators/corepressors. Interestingly, when we analyzed the predicted 3D NPAS4 model (see Fig. 7*B*) in the context of the distribution of NLSs and NESs, we observed that NLS-1 and NES-1 in the bHLH domain were easily accessible for interaction with the partners. In contrast, other signals were located in areas that can exist in multiple conformations adopted by the disordered NPAS4 C terminus and, in this way, influence the exposure and accessibility of signals in the C-terminal part of the protein. Additionally, Fig. 7*B* presents the unique mammalian NPAS4 sequence rich in PG and PR repeats, in which we predicted Pro^201^ (probability 0.85) as the site of hydroxylation. Interestingly, this sequence in the 3D model is located as a linker between the N-terminal and C-terminal structural parts of NPAS4 and is highly exposed. We hypothesize that this site might serve as a site for hydroxylation and subsequent ubiquitin-mediated degradation.

IDRs are known targets of intensive posttranslational modifications (PTM). Modifications such as phosphorylation, especially near the NLS or the NES, have been shown to regulate the intracellular distribution of proteins by activating or deactivating the localization motifs (32). We performed predictions for NPAS4 phosphorylation sites and found that 62 serine residues of NPAS4 are phosphorylated with a probability higher than 0.9. Some of the predicted serine residues were found in the NLSs or NESs, and some were found to be located in close proximity to these motifs: Ser^156^, Ser^161^ (NLS-2), Ser^233^, Ser^235^, Ser^245^ (NES-2), Ser^302^, Ser^317^ (NLS-3), and Ser^615^ (NLS-4); this suggests that phosphorylation is an influencing factor of NPAS4 localization. Choy *et al.* (33) suggest that NPAS4 in neuronal cells is phosphorylated by a member of the MAPK family. Interestingly, Western blot analysis showed the presence of dual bands for expressed YFP-NPAS4/277–340 (the area comprising NLS-3) and YFP-NPAS4/581–650 (the area comprising NES-4 and NLS-4) (Fig. S1*B*), which additionally supports our hypothesis regarding the role of PTMs.

We observed that NPAS4 is localized in the nucleus or the cytoplasm of COS-7 and N2a cells. The proportion of nuclear to cytoplasmic NPAS4 depended on the glucose concentration in the medium. Furthermore, cytoplasmic localization of NPAS4 was LMB-sensitive. The reasons behind these differences in results obtained from experiments of full-length NPAS4 performed in media with different concentrations of glucose remain unknown. The low-glucose medium used in our experiments (4.5 mm) corresponded to the concentration of glucose in normal metabolism, whereas the high-glucose concentration (25 mm) corresponded to diabetes. NPAS4 was shown to be one of the immediate early genes (IEG) that activate mechanisms related to the first defense against many cellular stresses (34). Interestingly, immediate early genes are regulated by a specific stimulus without *de novo* synthesis protein (35). Shamloo *et al.* (20) propose that cytoplasmic NPAS4 in brain cells serve as a source for translocation to other cellular compartments after ischemia, a finding that could suggest a role for NPAS4 shuttling in cells. Previous studies have demonstrated that glucose depolarization rapidly induces NPAS4 expression in murine and human pancreatic islets (11). Additionally, NPAS4 has been shown to be indispensable for the depolarization-dependent induction of almost 300 genes in the brain. Interestingly, for some of these genes, NPAS4 appears to be a dual regulator of transcription, with the ability to both induce and repress downstream gene expression (36). Odor-induced expression of NPAS4 in the olfactory bulb suppresses E3 ubiquitin-protein ligase Mdm2 expression (37). Additionally, in pancreatic cells, NPAS4 inhibits insulin gene activation (7).

As transcription factors, NPAS4 should be located in the nucleus to control gene transcription; however, the exclusion of transcription factors from the nucleus is a known mechanism that regulates their activity. For example, the regulation of HIF-1α includes compartmentalization of proteasome-dependent degradation activity and subcellular distribution of this protein (25). HIF-1α contains one NLS in its bHLH domain and one NLS in its C terminus, which is responsible for transporting HIF-1a to the nucleus when exposed to hypoxia (38). NPAS4 as a heterodimer with ARNT2 has been shown to activate the transcription of brain-derived neurotrophic factor (BDNF) (39). As ARNT2 is a strictly nuclear protein (40), the regulation of the nuclear localization of NPAS4 could be the limiting factor in transcription activation of BDNF, homeostatic genes in neurons, and other downstream targets such as VE-cadherin in endothelial cells (8).

The role of NPAS4 in many different cellular processes suggests additional roles for the cytoplasmic localization of this protein. NPAS4 recently was shown to induce autophagy in rat primary cortical neurons and to degrade Tau proteins involved in the pathogenesis of Alzheimer's disease and other tauopathies through an unknown mechanism (13). It was also recently documented that MAGED1 interacts with NPAS4 and promotes the activation of reporter genes. Interestingly, MAGED1 (a cytoplasmic protein) does not compete with ARNT or ARNT2 (nuclear proteins). It is proposed that the interaction between MAGED1 and the activated bHLH-PAS factor occurs prior to NPAS4 nuclear import and indirectly affects protein activity, probably via PTMs (21).

Our data indicate that glucose concentration could be one of the factors influencing NPAS4 localization. One of the posttranslational modifications initiated by high-glucose concentration is the enhanced protein *O*-glycosylation on Ser or Thr residues. This PTM can enhance glucotoxicity by impairing protein function and has been demonstrated to be involved in the insulin resistance and pathogenesis of diabetes (41). Interestingly, the prediction results for NPAS4 revealed a high probability of 0.8 for the *O*-glycosylation of Ser^4^ (bHLH), a 0.72 probability for Thr^333^ (PAS-2), and a >0.9 probability for many Ser and Thr residues in the C-terminal part of the protein, pointing to the possibility that these PTMs contribute to the regulation of NPAS4 localization and activity.

Herein, we have presented the first study of the presence of NoLS, NLS, and NES sequences in NPAS4. The alignment of NPAS4 amino acid sequences from *Rattus norvegicus* and *Homo sapiens* shows very high conservation, especially in the regions of the detected NLSs and NESs, indicating their importance for protein functionality (31). We show that NPAS4 is a shuttling protein with a very complicated and precise regulation of subcellular localization. The disordered character of the NPAS4 C terminus may serve as a platform for simultaneous interactions with many partners; this method of interaction may be responsible for the very precise and multifactor-dependent regulation of NPAS4 trafficking and may be crucial for the ability of NPAS4 to act as a biological sensor and switch to modulate the cross-talk of different signaling pathways. Our data indicate that glucose concentration could be one factor influencing NPAS4 localization by regulating NPAS4 activity as a transcription and cytoprotective factor. We hypothesize that the activation of NPAS4 transcription activity is dependent on its localization, ligand binding, and PTMs. Additional, more detailed studies are necessary to fully elucidate the regulation and different functions of NPAS4 in the cytosol, nucleus and other possible cellular compartments.

## Experimental procedures

### Plasmid construction

NPAS4 cDNA from *R. norvegicus* was a kind gift from Prof. Michael E. Greenberg (Harvard Medical School, Boston). Full-length cDNA encoding amino acid residues 1–802 was amplified by PCR and cloned into the EcoRI and SalI restriction sites of the MCS of the pEYFP-C1 vector (Clontech). Deletion mutants of NPAS4 were similarly cloned into the pEYFP-C1 vector. The point mutants YFP-NPAS4/1–40/R21A/K24A, YFP-NPAS4/26–59/L27A/L29A, YFP-NPAS4/145–194/R159A/K165A, YFP-NPAS4/195–276/L230A/I232A/L237A, YFP-NPAS4/277–340/R285A/K289A, and YFP-NPAS4/581–650/L591A/L594A/L598A were obtained by PCR-mediated site-directed mutagenesis as described by Ko and Ma (42) and cloned with LguI, EcoRI, and SalI restriction enzymes. All constructs were verified by DNA sequencing.

### Cell culture and DNA transfection

African green monkey kidney fibroblasts COS-7 (ATCC CRL-1651) and mouse albino neuroblastoma N2a cells (ECACC, Sigma-Aldrich) were cultured in DMEM with 1.0 or 4.5 g/liter glucose and l-glutamine (Lonza). The culture medium was supplemented with 10% fetal calf serum (FCS). Cells were cultured at 37 °C in a 95% air, 5% CO_2_ atmosphere. Cells were transfected with 3 μg of DNA/150,000 cells using Xfect^TM^ transfection reagent (Takara Clontech) according to the manufacturer's instructions. LMB (Sigma) was dissolved in 70% methanol at a concentration of 10^−3^ m and added to the medium during transfection at a final concentration of 10^−6^ m.

### Electrophoresis and Western blot analysis

To confirm the expression of NPAS4 and NPAS4 mutants in cultured cells in parallel with the microscopy experiments, SDS-PAGE and Western blot analysis were performed (Fig. S1). Samples of total protein extract were prepared by replacing the cell medium 24 h after transfection (after washing cells with PBS) with 2× SDS gel–loading buffer (43), transferring extracts to Eppendorf tubes, boiling samples for 5 min, and then centrifuging samples for 5 min (13,000 × *g*). Proteins were separated by 10% SDS-PAGE and transferred to a Whatman Protran nitrocellulose transfer membrane (Protran BA85, Schleicher & Schuell Pure, Sigma-Aldrich) with a mini Trans-Blot apparatus (Bio-Rad). The membrane was blocked at room temperature for 1 h in 3% milk blocking buffer (milk powder, Milchpulver, blotting grade, Roth) prepared in PBS supplemented with 0.2% Tween 20 (Sigma). Next, the membrane was incubated overnight at 4 °C with anti-GFP polyclonal antibodies (Clontech) (diluted 1:300 with milk buffer), which cross-react with YFP. Secondary goat anti-rabbit antibodies coupled to horseradish peroxidase (Vector Laboratories) were added (1:10,000 with milk buffer) and incubated at room temperature for 2 h. Blots were developed using Pierce ECL Plus Western blotting substrate (Thermo Scientific) according to the manufacturer's manual.

### Microscopy imaging

As described previously (44), prior to confocal microscopy imaging experiments, cells were seeded onto 0.17-mm–thick round glass coverslips (Menzel) and submerged in culture medium in 2-cm–diameter Petri dishes. Next, 20–24 h after transfection, the coverslips with the cell cultures were transferred onto a steel holder and mounted on a microscope stage. For COS-7 cells, the standard culture medium was replaced with 1 ml of DMEM/F12 without phenol red, buffered with 15 mm HEPES (Sigma), and supplemented with 2% fetal bovine serum (FBS, Sigma). Next, 0.25 μl of Draq5 DNA dye (BioStatus) was added to the cells for DNA visualization. During microscopy, the cell culture temperature was stabilized at 37 °C using a microincubator (Life Imaging Services, Box & Cube). Images of fluorescently labeled proteins were acquired using a Leica TCS SP5 II confocal system equipped with argon and helium neon lasers and a 63× oil objective lens (numerical aperture 1.4). YFP was excited using 514 nm light, and emitted fluorescence was observed in the range of 525 to 600 nm. The excitation wavelength of Draq5 was 633 nm, and emission was detected at 650 to 800 nm. Gamma correction of 0.6 was set for images with Draq5 dye. Fluorescence microscopy was performed in 2-cm–diameter Petri dishes in low- or high-glucose DMEM using an Olympus IX71 microscope with a YFP filter at 24 or 48 h after transfection. At least 50 cells were observed for each cDNA construct in one experiment, and at least three independent experiments were performed for each cDNA construct. Images are presented for representative cells with a typical phenotype characteristic of more than 95% of the cells. For those cells presenting many phenotypes, representative images of all cell phenotypes are presented.

### In silico analysis of the NPAS4 sequence

PSIPRED (Protein Structure Prediction Server) (45) was used to predict the secondary structure of NPAS4. NPAS4 domain architecture was predicted using SMART (simple modular architecture tool (46), http://smart.embl-heidelberg.de)^3^ and PROSITE (a database of protein families and domains, http://expasy.org/tools/scanprosite/). Sequence alignments were obtained using ClustalX (47) (http://www.clustal.org/). Predictions for potential NLS sequences were performed using NucPred (48), PSORTII (49) (http://www.psort.org/), cNLS Mapper, and NLStradamus (50) (http://www.moseslab.csb.utoronto.ca/NLStradamus/). Predictions of potential NoLS motifs were performed using NoD (nucleolar localization sequence detector, http://www.compbio.dundee.ac.uk/www-nod/) (51, 52, 63, 64). Predictions of potential NES sequences were performed using NetNes 1.1 server (53) (http://www.cbs.dtu.dk/services/NetNES/); ValidNES (54) http://validness.ym.edu.tw); NES Finder 0.2 (http://research.nki.nl/fornerodlab/NES-Finder.htm); and LocNES (55) (http://prodata.swmed.edu/LocNES/LocNES.php). Predictions of NPAS4 phosphorylation sites were performed using KinasePhos2.0 (56) (http://kinasephos2.mbc.nctu.edu.tw/); and 9aaTAD predictions were performed using the 9aaTAD prediction tool (moderately stringent patterns were used, which are known to be best for mammalian transcription factors (30)) (http://www.med.muni.cz/9aaTAD/). Predictions of protein disorder were performed using VL3 predictor of intrinsically disordered regions (57, 58) (http://www.dabi.temple.edu/disprot/predictor.php). The NPAS4 rat model was generated using Phyre 2 (59). Residues 1–340 encompassing the bHLH and PAS domains of NPAS4 were modeled with confidence higher than 90% using the following templates: c4f31B, c4zprB, C4f31A, c4zp4D, c5sy5B, and c5sy7B. C-terminal regions of NPAS4 lacking any template and predicted as disordered were modeled *ab initio*, which was highly unreliable. Ligand binding sites were predicted using the 3DLigandSite Web server (60) (http://www.sbg.bio.ic.ac.uk/3dligandsite) for the Phyre2-generated model of NPAS4 residues 1–340.

Predictions of *O*-glycosylation sites were performed using the NetOGlyc 4.0 server (61) (http://www.cbs.dtu.dk/services/NetOGlyc/). Predictions of hydroxylation sites were performed using PredHydroxy:Prediction of Protein Hydroxylation site (62).

## Author contributions

B. G.-M. conceptualization; B. G.-M. data curation; B. G.-M. and A. O. formal analysis; B. G.-M., M. Z., and A. O. funding acquisition; B. G.-M. investigation; M. Z. help with confocal imaging; B. G.-M., M. Z., and A. O. writing-original draft; B. G.-M. project administration; B. G.-M., M. Z., and A. O. writing-review and editing.

## Supplementary Material

Supporting Information

## Footnotes

3 Please note that the JBC is not responsible for the long-term archiving and maintenance of this site or any other third party–hosted site.

## Footnotes

2 The abbreviations used are:

NPAS4: neuronal Per-Arnt-Sim domain–containing protein 4
bHLH: basic helix–loop–helix
PAS: Per-Arnt-Sim
HIF: hypoxia-inducible factor
ARNT: aryl hydrocarbon nuclear translocator
NLS: nuclear localization signal
NES: nuclear export signal
LMB: leptomycin B
YFP: yellow fluorescent protein
DMEM: Dulbecco's modified Eagle's medium
aa: amino acid(s)
NoLS: nucleolar localization signal
NoD: nucleolar localization sequence detector
PAC: PAS domain-associated C terminus
IDR: intrinsically disordered region
PTM: posttranslational modification
N2a: Neuro 2a cells.
