# Supplementary material for: Multiple sequences orchestrate subcellular trafficking of neuronal PAS domain–containing protein 4 (NPAS4)
Source: J Biol Chem. 2018 Jun 13;293(29):11255–70. doi: 10.1074/jbc.RA118.001812 (PMC6065191; doi:10.1074/jbc.RA118.001812)
Supplement: Supporting Information [file supp_293_29_11255__index.html]

Multiple sequences orchestrate subcellular trafficking of neuronal PAS domain-containing protein 4 (NPAS4) — Multiple sequences regulate NPAS4 shuttling — Multiple sequences orchestrate subcellular trafficking of neuronal PAS domain–containing protein 4 (NPAS4) — Multiple sequences regulate NPAS4 shuttling — Supporting Information 

# Multiple sequences orchestrate subcellular trafficking of neuronal PAS domain–containing protein 4 (NPAS4)

## Supporting Information

- Multiple sequences orchestrate subcellular trafficking of neuronal PAS domain-containing protein 4 (NPAS4) - Supporting Figures
