# Supplementary material for: Multiple sequences orchestrate subcellular trafficking of neuronal PAS domain–containing protein 4 (NPAS4)
Source: J Biol Chem. 2018 Jun 13;293(29):11255–70. doi: 10.1074/jbc.RA118.001812 (PMC6065191; doi:10.1074/jbc.RA118.001812)
Supplement: Supporting Information [file supp_RA118.001812_135162_2_supp_149681_p9vc25.docx]

**Multiple sequences orchestrate subcellular trafficking of neuronal PAS domain-containing protein 4 (NPAS4)**

Beata Greb-Markiewicz, Mirosław Zarębski, Andrzej Ożyhar

**Supporting Information:**

**Figure S1.** Western blot analysis of expression of YFP tagged NPAS4 and NPAS4 derivatives in transfected COS-7 cells

**Figure S2.** Subcellular distribution of full-length NPAS4 in COS-7 and N2a cells

**Figure S3.** Influence of LMB and methanol on subcellular distribution of NPAS4 in COS-7 and N2a cells in high glucose medium

**Figure S4.** Subcellular distribution of the NPAS4 bHLH and PAS domain derivatives in COS-7 and N2a cells in high glucose medium.

**Figure S5.** Subcellular distribution of the bHLH domain of NPAS4 derivatives in COS-7 and N2a cells in high glucose medium

**Figure S6.** Subcellular distribution of the C-terminal part of NPAS4 *short* derivatives in COS-7 and N2a cells in high glucose medium

**Figure S7.** Subcellular distribution of NPAS4 C-terminal *combined* derivatives in COS-7 and N2a cells in high glucose medium

**Figure S8.** Subcellular distribution of NPAS4 derivatives in COS-7 and N2a cells

**Figure S9.** Subcellular distribution of N-terminal NPAS4 derivatives in COS-7 and N2a cells in high glucose medium

**Figure S10.** Subcellular distribution of NPAS4 derivatives in COS-7 and N2a cells

**Figure S1**

**
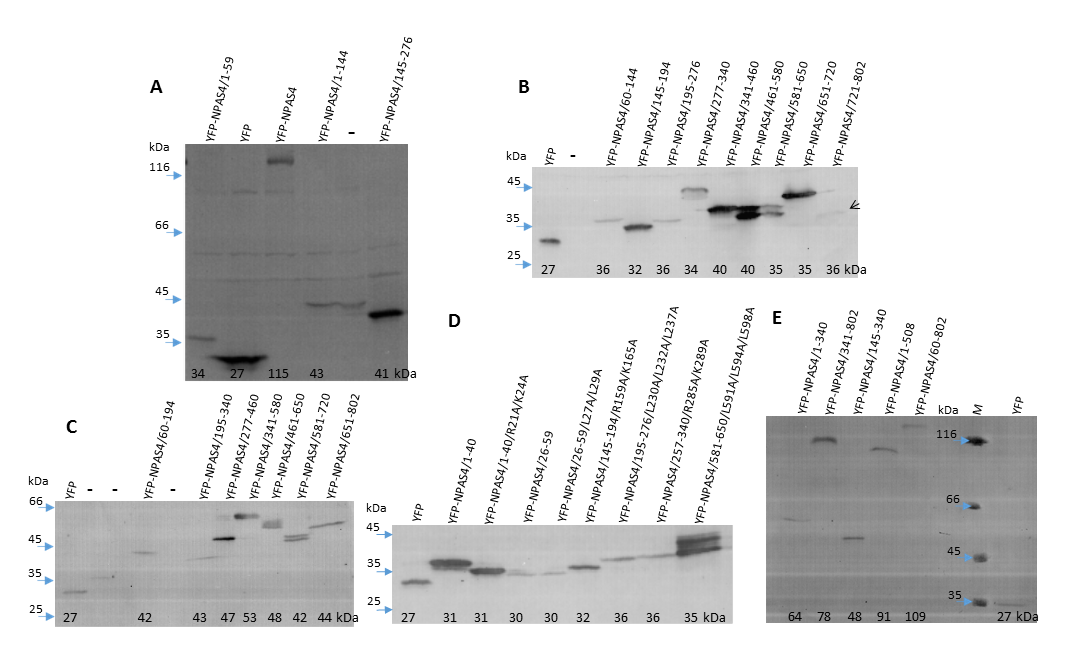
**

**Figure S1.** Western blot analysis of expression of YFP-tagged NPAS4 and NPAS4 derivatives in transfected COS-7 cells. Detection with anti-GFP antibodies. The theoretical molecular mass of proteins are presented on the bottom of the images. (A) Expression of *short* NPAS4 fragments tagged with YFP, YFP as a control. (B) Expression of *long* NPAS4 fragments tagged with YFP, with YFP as a control. (C) Expression of *short* NPAS4 fragments tagged with YFP (YFP-NPAS4/1-59), full-length NPAS4 tagged with YFP, *long* NPAS4 fragments tagged with YFP (YFP-NPAS4/1-144, YFP-NPAS4/145-276), and YFP as controls. (D) Expression of NPAS4 bHLH domain fragments (YFP-NPAS4/1-40, YFP-NPAS4/26-59) and point mutants of NPAS4 tagged with YFP, with YFP as a control. (E) Expression of NPAS4 fragments tagged with YFP (YFP-NPAS4/1-340, YFP-NPAS4/341-802, YFP-NPAS4/145-340, YFP-NPAS4/1-508, YFP-NPAS4/60-802), with YFP as a control. M, protein marker.

**Figure S2**


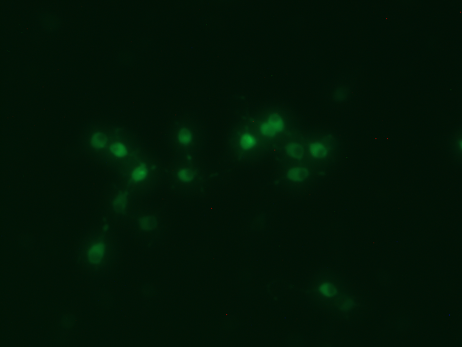

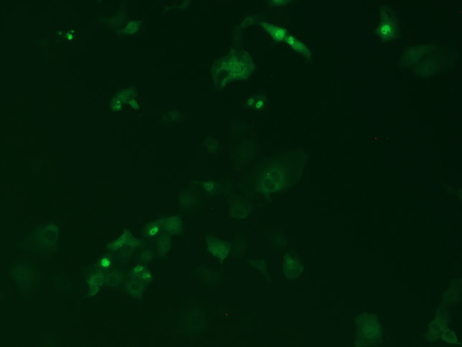

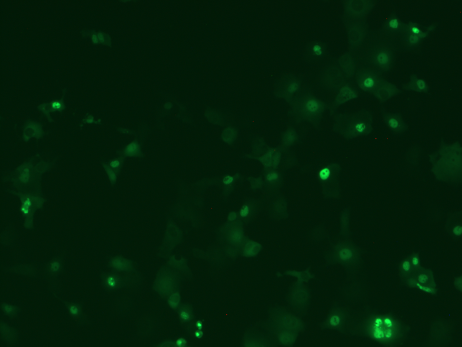

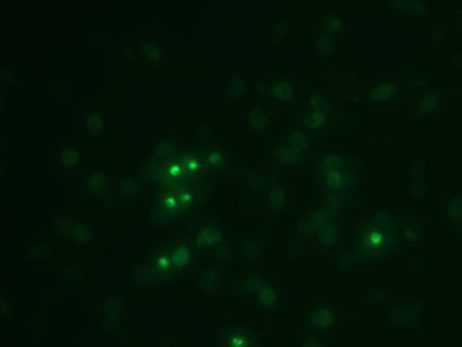

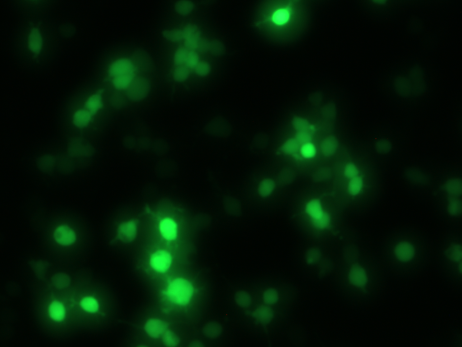


YFP


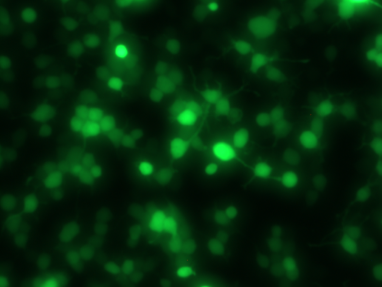

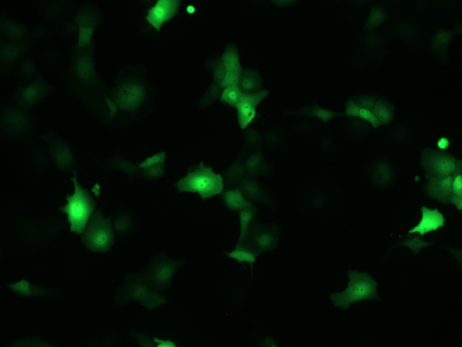

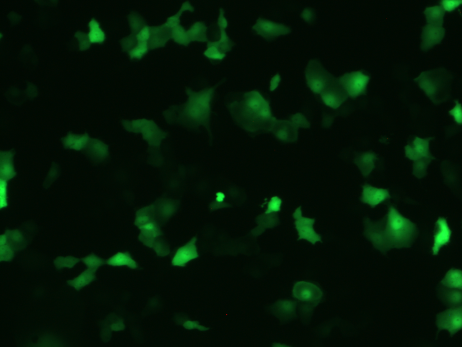


B

COS-7

Neuro 2a

24h

48h

YFP

YFP


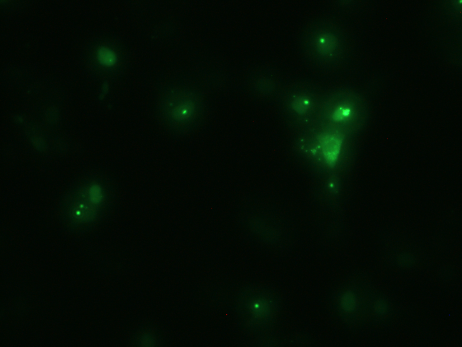

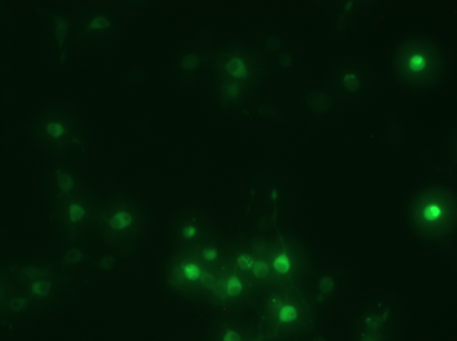


A

COS-7

Neuro 2a

YFP-NPAS4

24h

48h

YFP-NPAS4

High-glucose

DMEM

Low-glucose

DMEM

24h

**Figure S2.** Subcellular distribution of full-length NPAS4 in COS-7 and N2a cells. Subcellular localizations of YFP-tagged full-length NPAS4 and YFP were analysed by fluorescence microscopy. A minimum of 50 cells were counted to quantify the percentage of cells with nuclear (N)/cytoplasmic (C) localization in a minimum of 3 independent experiments.

(A) Representative images of the subcellular distribution of the YFP-tagged NPAS4 24 h (low-and high-glucose DMEM and 48 h after transfecting COS-7 and Neuro 2a cells (high-glucose DMEM). (B) Representative images of the subcellular distribution of YFP 24 h and 48 h after transfecting COS-7 and Neuro 2a cells in high-glucose DMEM.

**Figure S3**


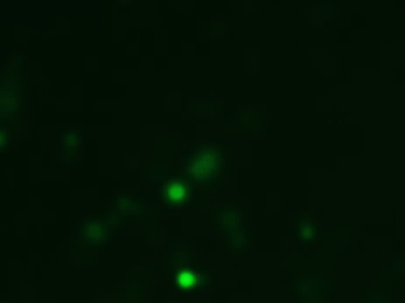

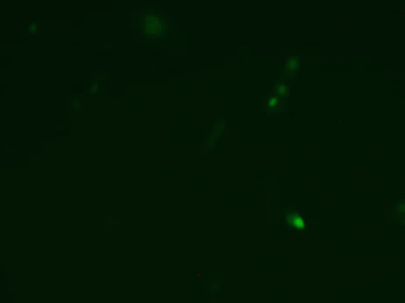

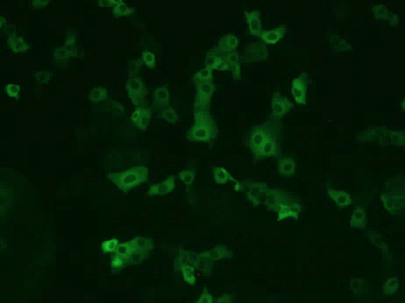

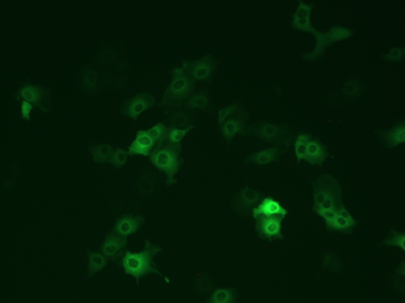


COS-7


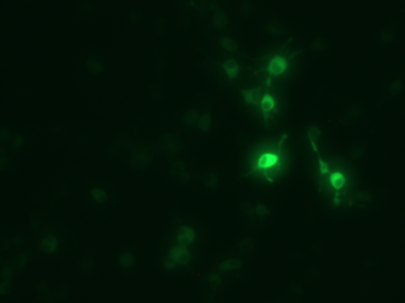


+ Methanol


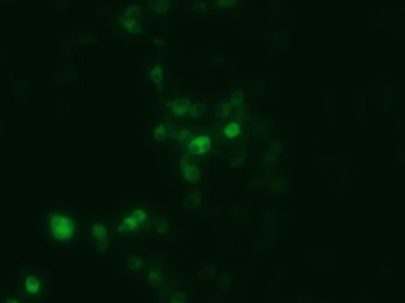


YFP-NPAS4/461-650


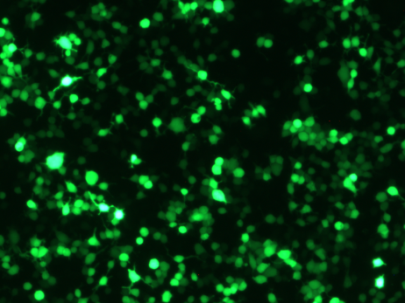

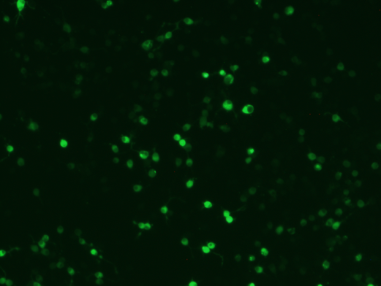

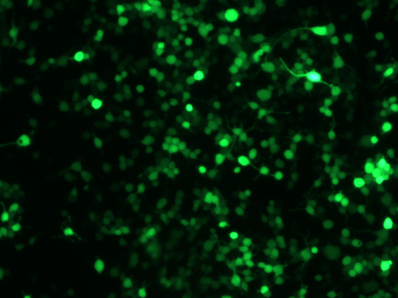

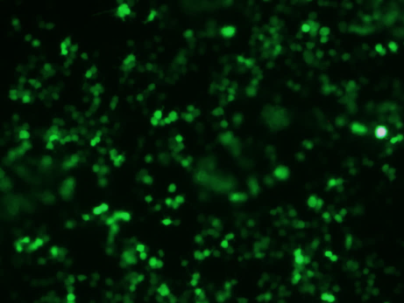


Neuro 2a

YFP

YFP

YFP

YFP-NPAS4

YFP-NPAS4

YFP-NPAS4

+ LMB

+ Methanol

A

YFP-NPAS4/461-650

YFP-NPAS4/461-650

YFP-NPAS4/461-650

YFP-NPAS4/461-650

YFP-NPAS4/461-650

+ Methanol

+ LMB

+ LMB

Neuro 2a


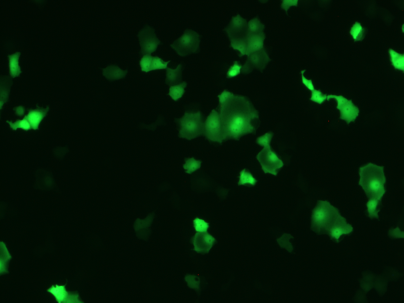

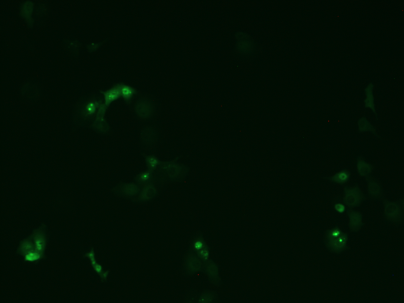

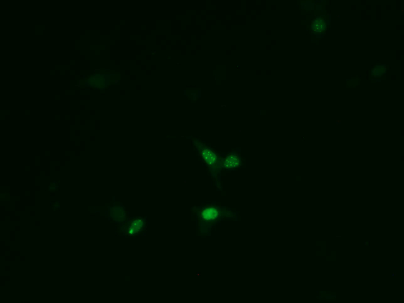

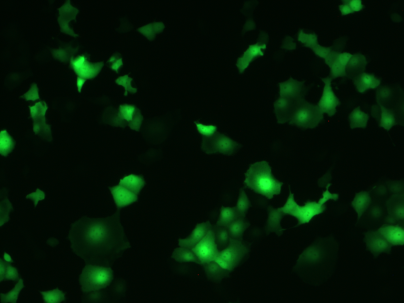


+ LMB


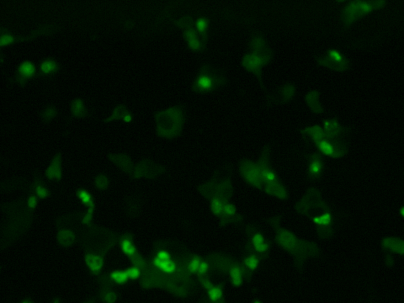


YFP-NPAS4

YFP-NPAS4

+ LMB

+ Methanol


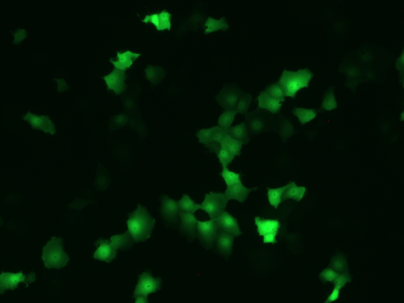


YFP

+ Methanol

YFP

YFP

YFP-NPAS4

COS-7

+ LMB


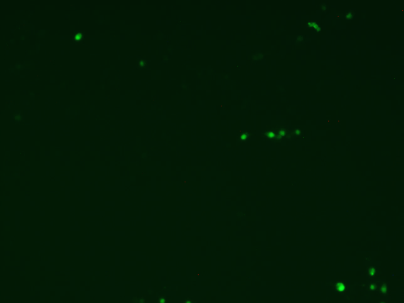


YFP-NPAS4

+ LMB


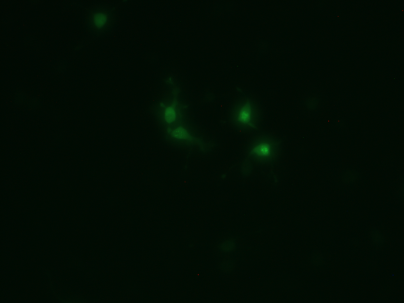


YFP-NPAS4

+ Methanol

B

**Figure S3**. Influence of LMB and methanol on the subcellular distribution of NPAS4 in COS-7 and N2a cells in high-glucose medium. Subcellular localizations of expressed YFP-tagged proteins were analysed by fluorescence microscopy 24 h after transfection. (A) Representative images of the subcellular distribution of YFP and YFP-tagged NPAS4 under normal conditions, after LMB addition, and after methanol addition in COS-7 and N2a cells. LMB and methanol (LMB solvent) had no influence on ubiquitous YFP distribution. The distribution of YFP-NPAS4 shifted to strictly nuclear localization after LMB addition, while no influence was observed for methanol as a control. (C) Representative images of the subcellular distribution of the YFP-tagged NPAS4/461-650 fragment under normal conditions, after LMB addition, and after methanol addition. LMB addition resulted in a shift from cytoplasmic to a primarily nuclear distribution of YFP-NPAS4/461-650, while no influence was observed for methanol as a control.

**Figure S4**


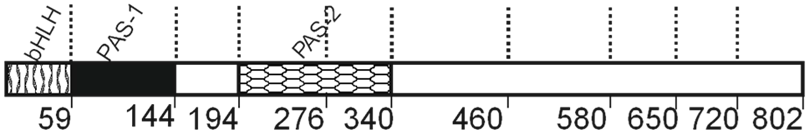

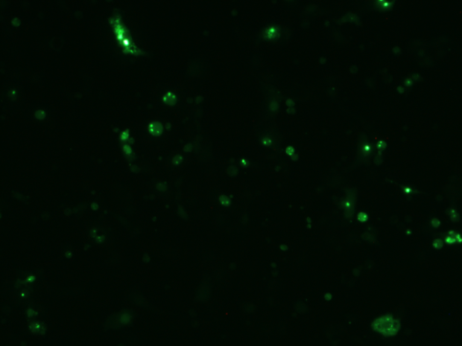

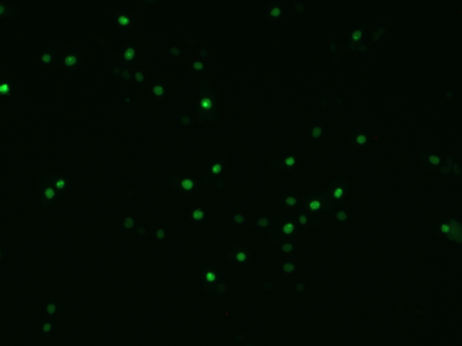

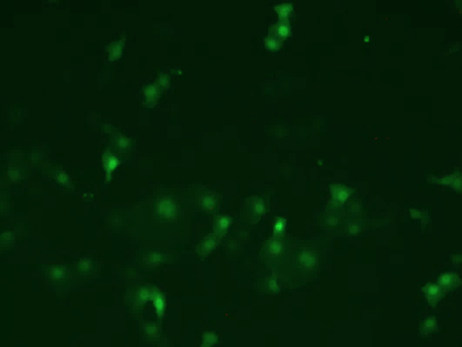

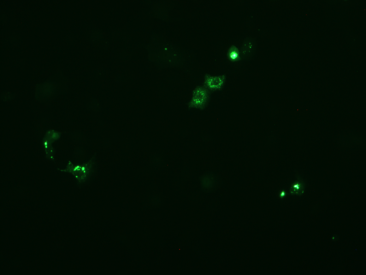

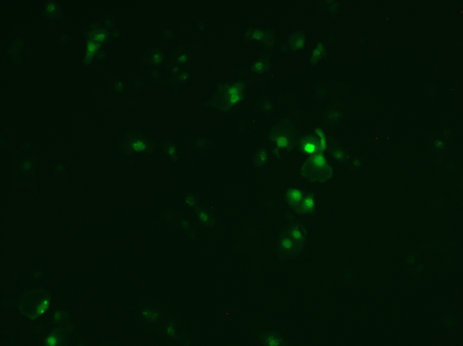

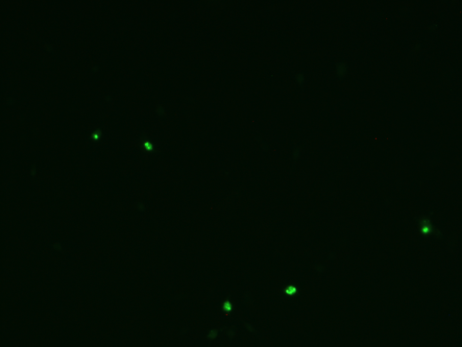

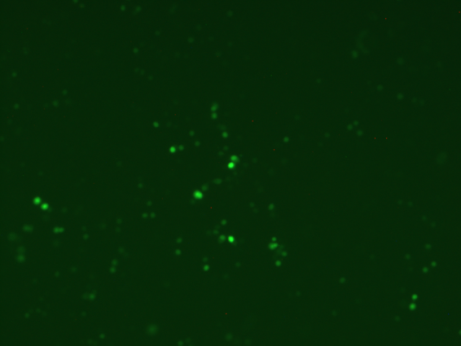

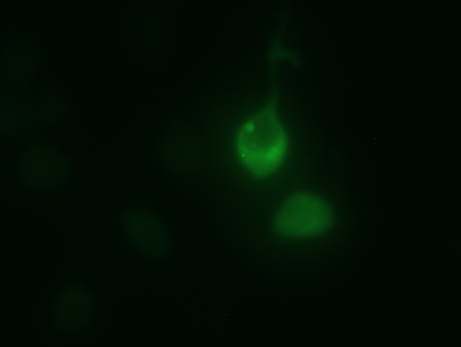

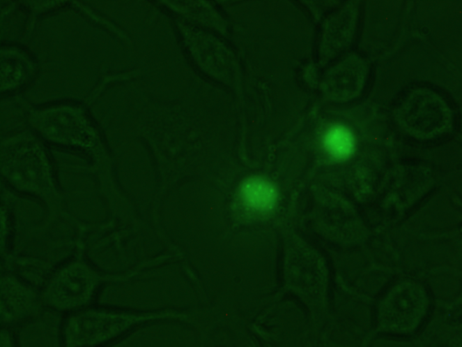

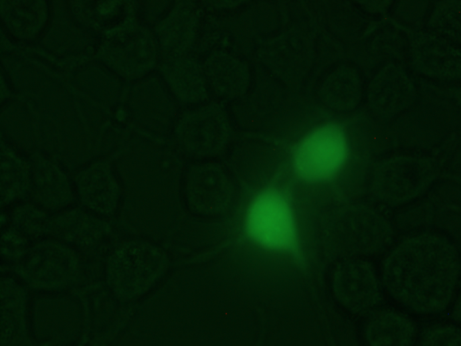

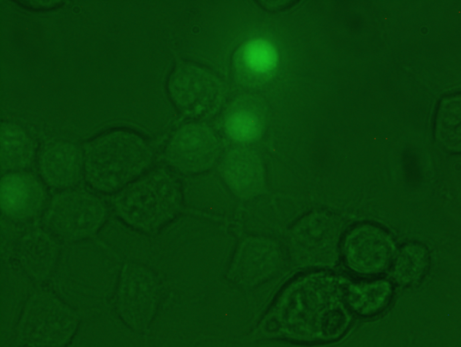

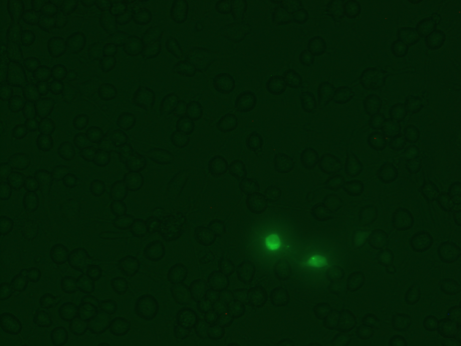

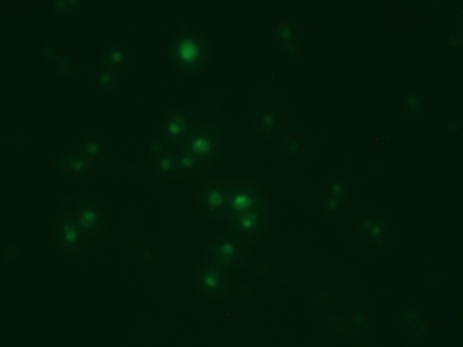

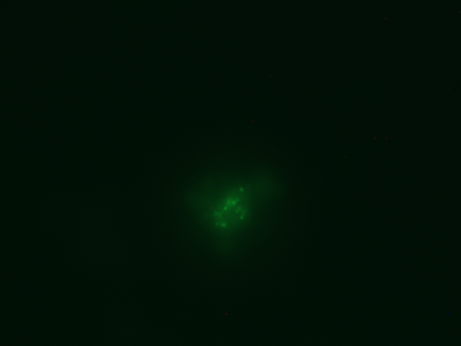


COS-7

Neuro 2a

YFP-NPAS4/1-59

YFP-NPAS4/60-144

YFP-NPAS4/145-194

YFP-NPAS4/195-276

YFP-NPAS4/277-340


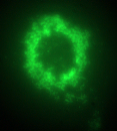

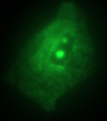

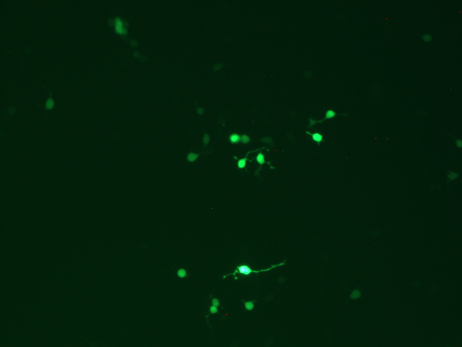


NPAS4

YFP-NPAS4/1-59

YFP-NPAS4/60-144

YFP-NPAS4/145-194

YFP-NPAS4/195-276

YFP-NPAS4/277-340

**Figure S4**. Subcellular distribution of the NPAS4 bHLH domain and PAS domain derivatives in COS-7 and N2a cells in high-glucose medium. Subcellular localizations of the expressed YFP-tagged proteins were analysed by fluorescence microscopy 24 h after transfection. Representative images of the subcellular distribution of the NPAS4 bHLH domain derivatives YFP-NPAS4/1-59, YFP-NPAS4/60-144, YFP-NPAS4/145-194, YFP-NPAS4/195-276 and YFP-NPAS4/277-340 in COS-7 (left panel) and Neuro 2a (right panel) cells. Schematic representation of NPAS4 protein is presented for clarity.

**Figure S5**


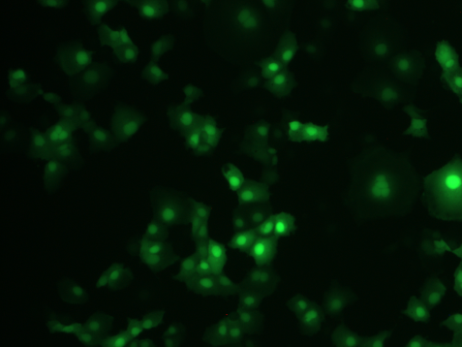

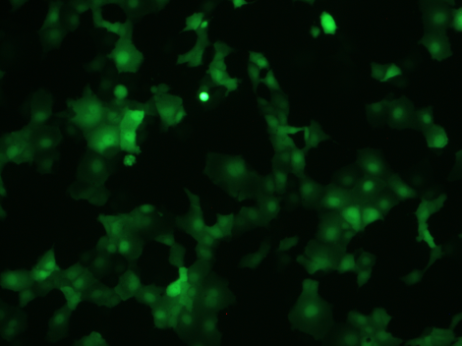

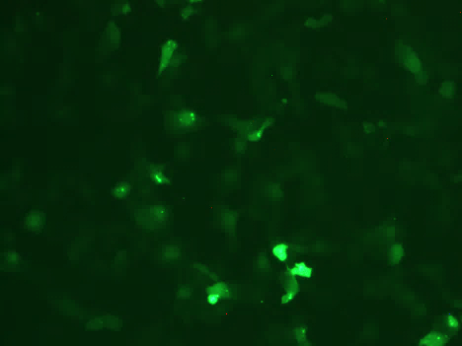

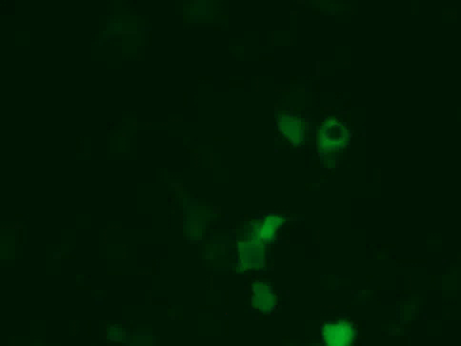

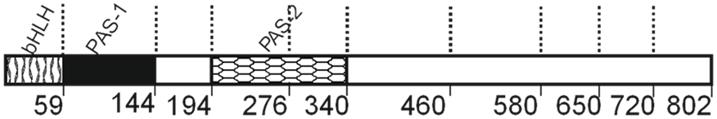

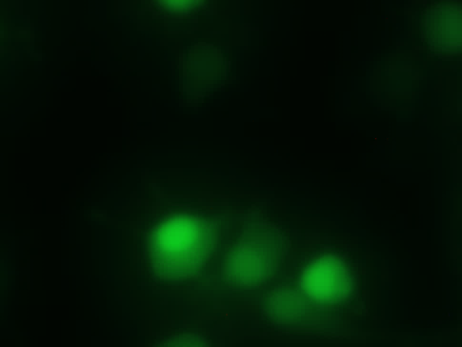

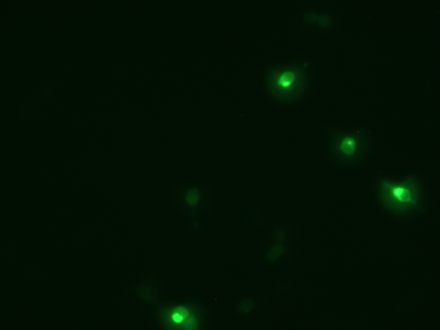

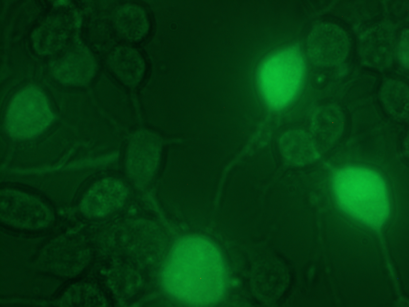

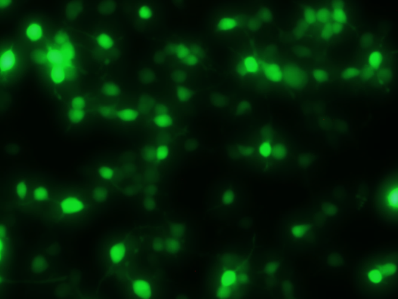

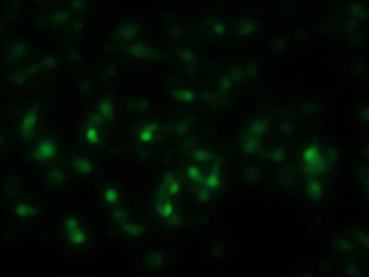

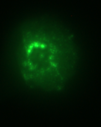

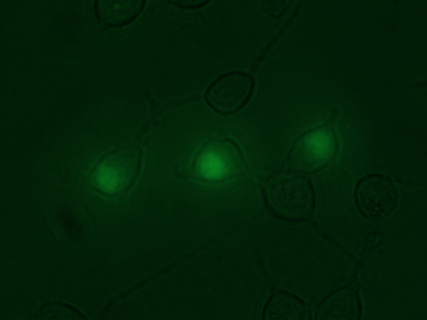

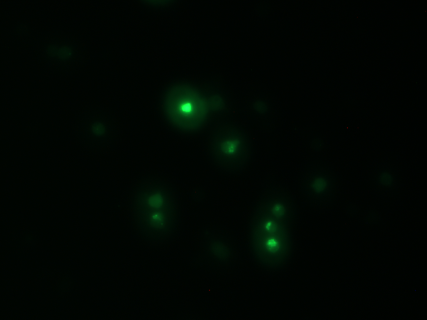

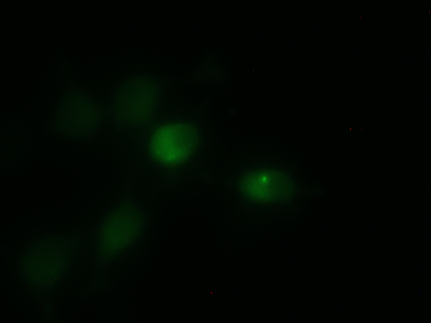


YFP-NPAS4/1-40

YFP-NPAS4/1-40/R21A/K24A

YFP-NPAS4/26-59

YFP-NPAS4/26-59/L27A/L29A

NPAS4

Neuro2a

COS-7

YFP-NPAS4/1-40

YFP-NPAS4/1-40/R21A/K24A

YFP-NPAS4/26-59

YFP-NPAS4/26-59/L27A/L29A

**S5.** Subcellular distribution of the bHLH domain of NPAS4 derivatives in COS-7 and N2a cells in high-glucose medium. Subcellular localizations of the expressed YFP-tagged proteins were analysed by fluorescence microscopy 24 h after transfection. Representative images of the subcellular distributions of YFP-NPAS4/1-40, YFP-NPAS4/1-40/R21A/K24A, YFP-NPAS4/26-59, YFP-NPAS4/26-59 and YFP-NPAS4/26-59/L27A/L29A in COS-7 (left panel) and Neuro 2a (right panel) cells. Schematic representation of NPAS4 protein is presented for clarity.

**Figure S6**


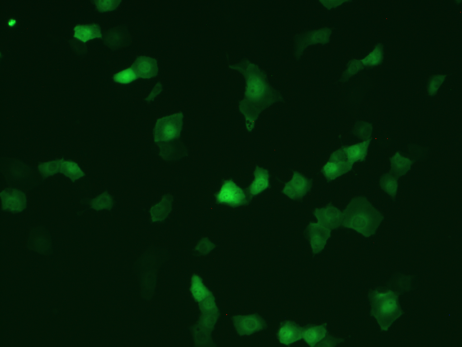

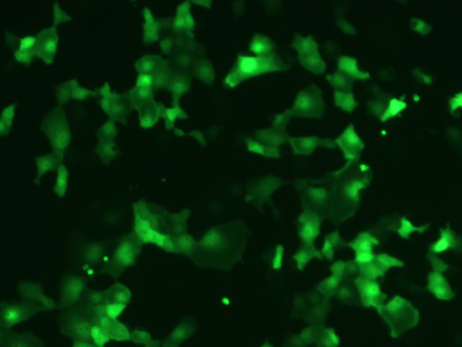

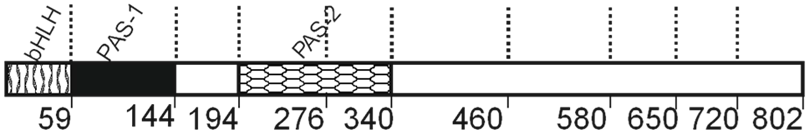

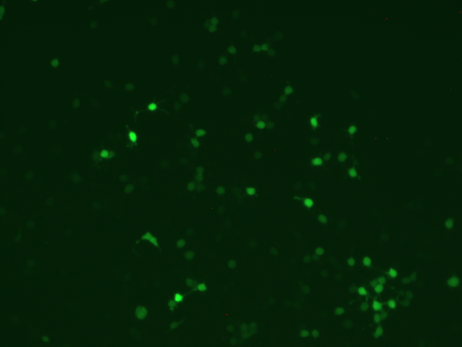

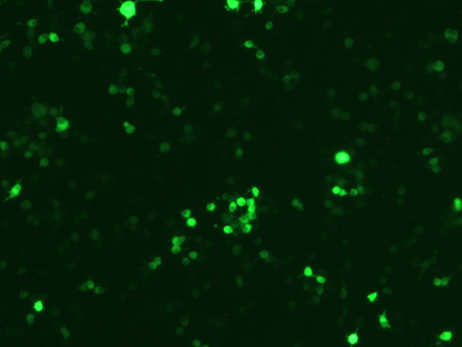

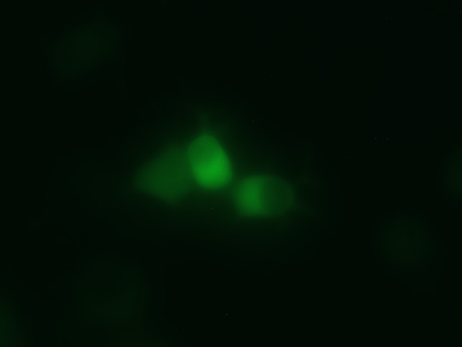

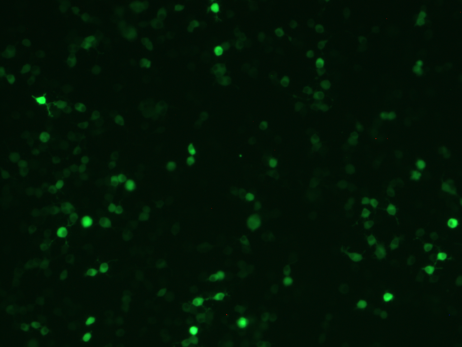

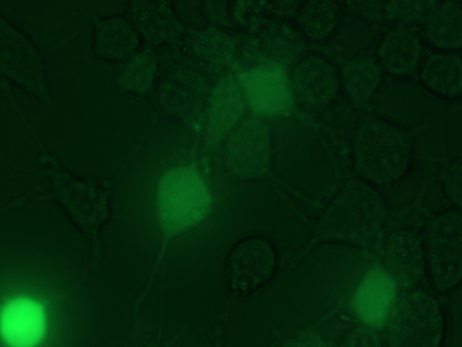

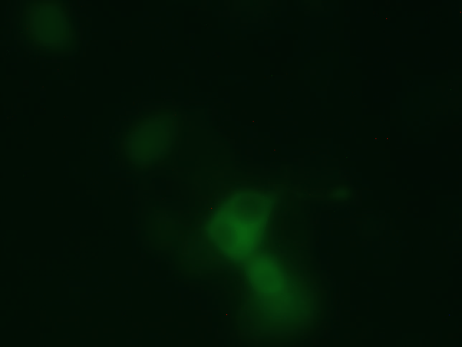

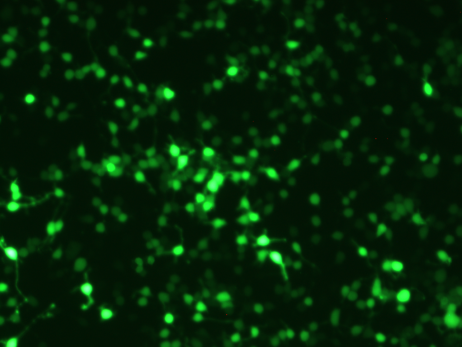

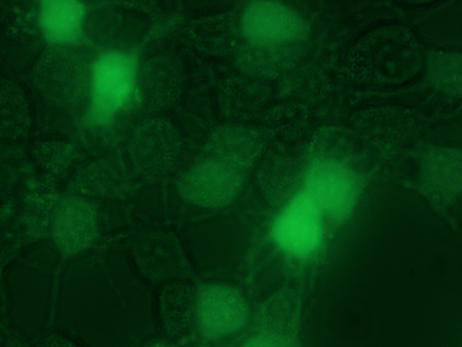

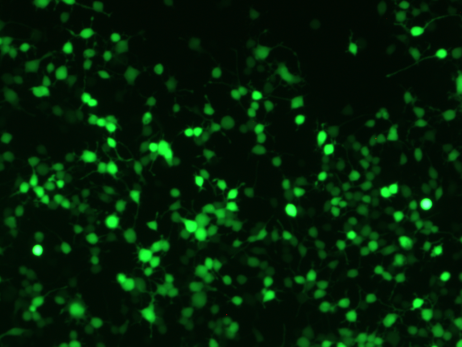

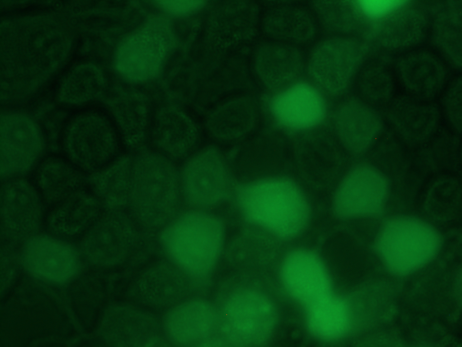

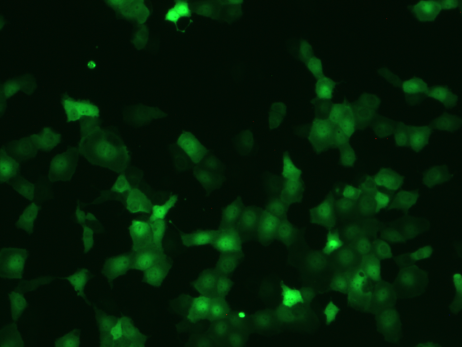

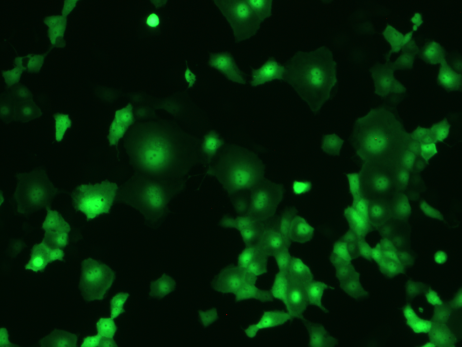

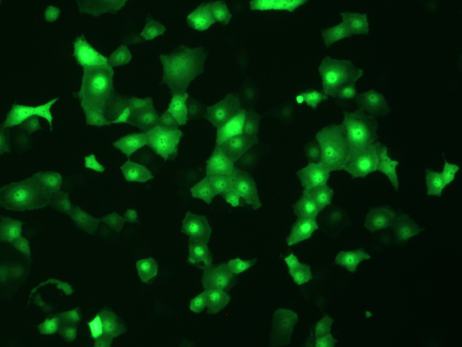


COS-7

Neuro 2a

YFP-NPAS4/341-460

YFP-NPAS4/581-650

YFP-NPAS4/651-720

YFP-NPAS4/721-802

NPAS4

YFP-NPAS4/461-580

YFP-NPAS4/341-460

YFP-NPAS4/461-580

YFP-NPAS4/341-460

YFP-NPAS4/721-802

YFP-NPAS4/651-720

YFP-NPAS4/581-650

**Figure S6.** Subcellular distribution of the C-terminal part of NPAS4 *short* derivatives in COS-7 and N2a cells in high-glucose medium. Subcellular localizations of the expressed YFP-tagged proteins were analysed by fluorescence microscopy 24 h after transfection. Representative images of the subcellular distributions of the NPAS4 C-terminal derivatives YFP-NPAS4/341-460, YFP-NPAS4/461-580, YFP-NPAS4/581-650, and YFP-NPAS4/651-720 and YFP-NPAS4/721-802 in COS-7 (left panel) and Neuro 2a (right panel) cells. Schematic representation of NPAS4 protein is presented for clarity.

**Figure S7**


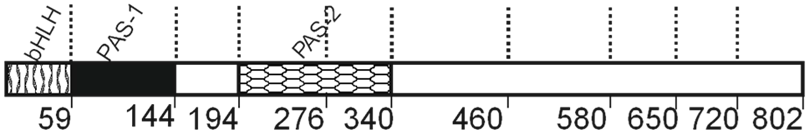

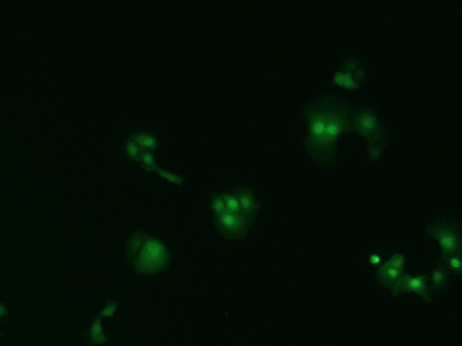

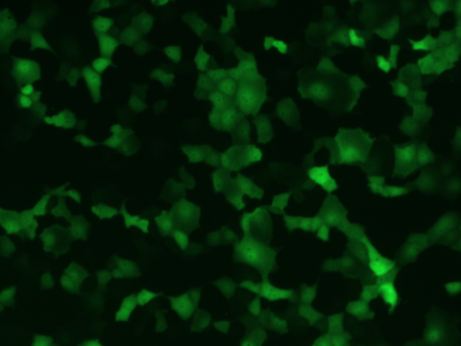

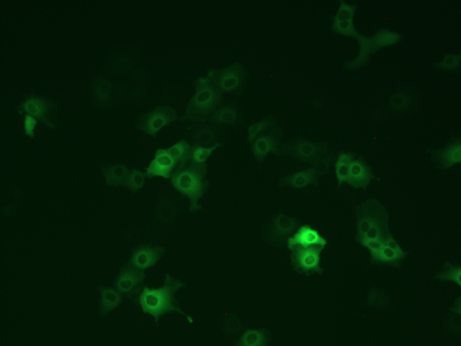

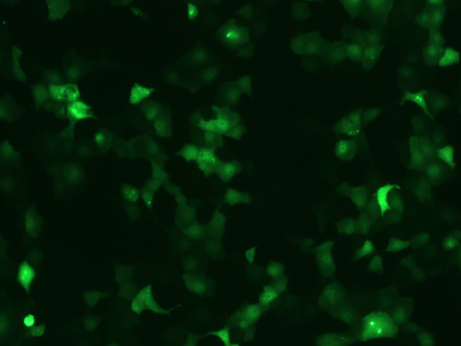

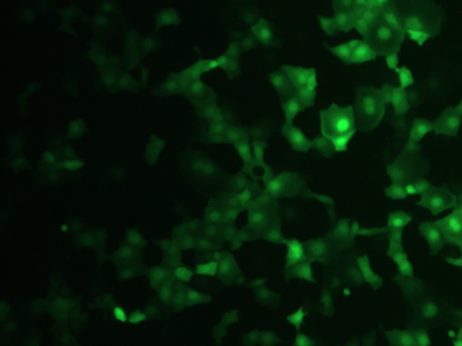

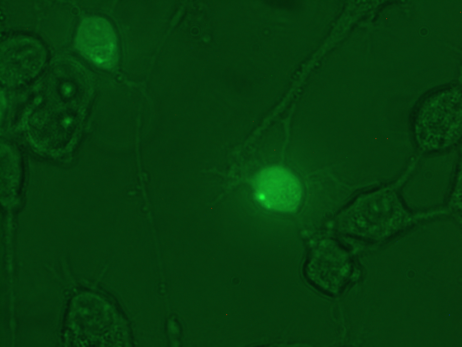

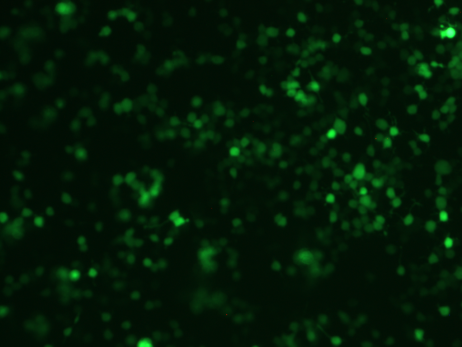

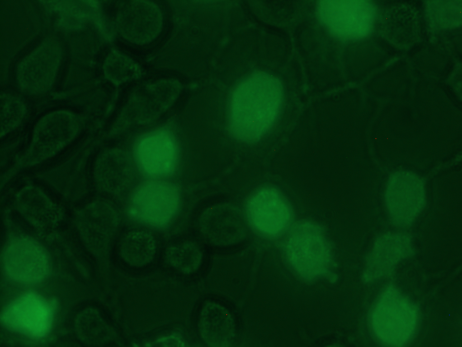

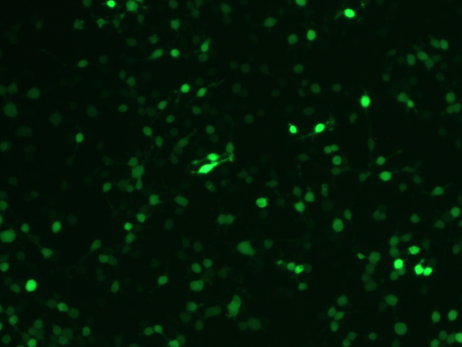

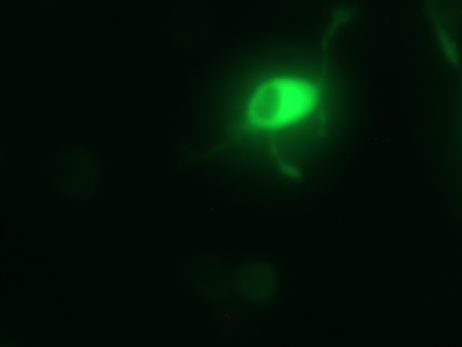

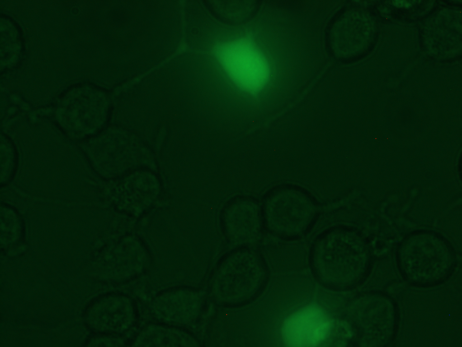

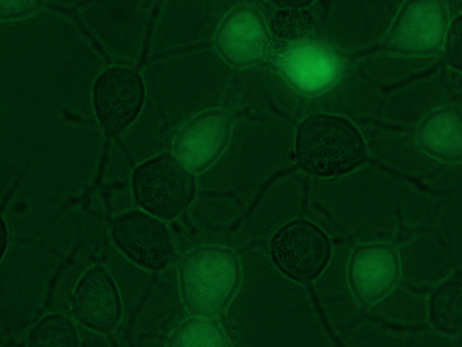

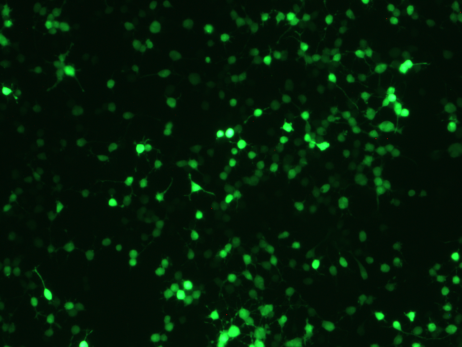


YFP-NPAS4/277-460

YFP-NPAS4/341-580

YFP-NPAS4/461-650

YFP-NPAS4/581-720

YFP-NPAS4/651-802

COS-7

Neuro 2a


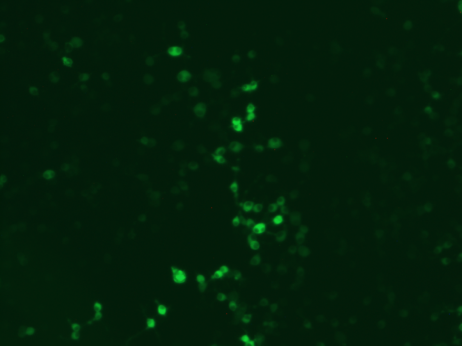

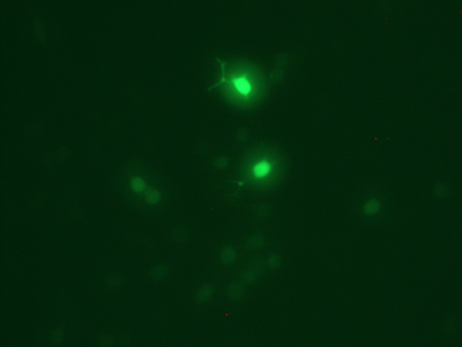


NPAS4

YFP-NPAS4/341-580

YFP-NPAS4/277-460

YFP-NPAS4/581-720

YFP-NPAS4/461-650

YFP-NPAS4/651-802

**Figure S7.** Subcellular distribution of NPAS4 C-terminal *combined* derivatives in COS-7 and N2a cells in high-glucose medium. Subcellular localizations of the expressed YFP-tagged proteins were analysed by fluorescence microscopy 24 h after transfection. Representative images of the subcellular distributions of the NPAS4 C-terminal derivatives YFP-NPAS4/277-460, YFP-NPAS4/341-580, YFP-NPAS4/461-650, YFP-NPAS4/581-720 and YFP-NPAS4/651-802 in COS-7 (left panel) and Neuro 2a (right panel) cells. The observed subcellular distributions of the NPAS4 derivatives in both cell lines are analogous. Schematic representation of NPAS4 protein is presented for clarity.

**Figure S8**


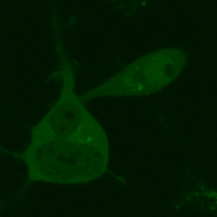

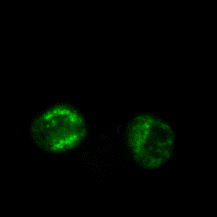

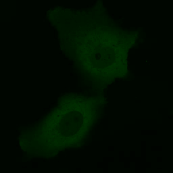

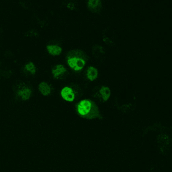

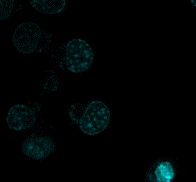

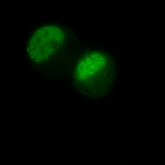

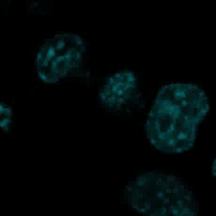


COS-7

Neuro 2a

+ Draq5

+ Draq5

YFP-NPAS4/145-277

YFP-NPAS4/195-340

YFP-NPAS4/1-340

YFP-NPAS4/145-340

YFP-NPAS4/341-802

YFP-NPAS4/1-580

YFP-NPAS4/60-802

YFP-NPAS4/145-277

YFP-NPAS4/195-340

YFP-NPAS4/145-340

YFP-NPAS4/1-340

YFP-NPAS4/1-580

YFP-NPAS4/341-802

YFP-NPAS4/60-802

BF

+ Draq5

BF

BF

merge

YFP-NPAS4/1-144

YFP-NPAS4/1-144

YFP-NPAS4/60-194

YFP-NPAS4/60-194

**Figure S8.** Subcellular distributions of NPAS4 derivatives in COS-7 and N2a cells. Subcellular localizations of the expressed YFP-tagged proteins were analysed by confocal fluorescence microscopy 24 h after transfecting COS-7 and Neuro 2a cells. Nuclei and nucleoli were stained with Draq5. Representative images (single confocal plane) of typical (presented by more than 95% of cells if not stated otherwise) subcellular distributions of the NPAS4 derivatives are presented: YFP-NPAS4/1-144, YFP-NPAS4/60-194, YFP-NPAS4/145-276, YFP-NPAS4/195-340, YFP-NPAS4/1-340, YFP-NPAS4/1-508 and YFP-NPAS4/60-802 in COS-7 (low glucose medium, left panel) and Neuro 2a (high glucose medium, right panel) cells. BF, bright field image, Bar, 10 µm.

**Figure S9**

YFP-NPAS4/1-144

YFP-NPAS4/60-194

YFP-NPAS4/145-276

YFP-NPAS4/195-340

COS-7

Neuro 2a

NPAS4

YFP-NPAS4/1-144

YFP-NPAS4/60-144

YFP-NPAS4/145-276

YFP-NPAS4/195-340

YFP-NPAS4/145-340

YFP-NPAS4/145-340

**Figure S9.** Subcellular distributions of N-terminal NPAS4 derivatives in COS-7 and N2a cells in high-glucose medium. Subcellular localizations of the expressed proteins were analysed by fluorescence microscopy 24 h after transfection. Representative images of the subcellular distributions of the NPAS4 N-terminal derivatives YFP-NPAS4/1-144, YFP-NPAS4/60-194, YFP-NPAS4/145-276, YFP-NPAS4/195-340 and YFP/NPAS4/145-340 in COS-7 (left panel) and Neuro 2a (right panel) cells are presented. Schematic representation of NPAS4 protein is presented for clarity.

**Figure S10**

A

B

COS-7

Neuro 2a

YFP-NPAS4/1-340

YFP-NPAS4/341-802

YFP-NPAS4/1-508

YFP-NPAS4/60-802

NPAS4

YFP-NPAS4/1-340

YFP-NPAS4/341-802

YFP-NPAS4/1-508

YFP-NPAS4/60-802

YFP-NPAS4/60-802

YFP-NPAS4/60-802

Low-glucose DMEM

**Figure S10.** Subcellular distribution of various NPAS4 derivatives in COS-7 and N2a cells. Subcellular localizations of the expressed YFP-tagged proteins were analysed by fluorescence microscopy 24 h after transfecting COS-7 and Neuro 2a cells. (A) Representative images of the subcellular distributions of the NPAS4 derivatives, YFP-NPAS4/1-340, YFP-NPAS4/1-508, YFP-NPAS4/341-802 and YFP-NPAS4/60-802 in COS-7 (left panel) and Neuro 2a (right panel) cells in high-glucose DMEM. (B) Representative images of the subcellular distributions of YFP-NPAS4/60-802 in COS-7 (left panel) and Neuro 2a (right panel) cells in low-glucose DMEM. Schematic representation of NPAS4 protein is presented for clarity.
